# Directed evolution of the pathogenic mold *Aspergillus fumigatus* reveals novel genes contributing to triazole resistance

**DOI:** 10.1128/aac.01635-25

**Published:** 2026-03-04

**Authors:** Mariana Handelman, Argha Sarkar, Avital Varshavsky, Yona Shadkchan, Xuefei Chen, Gerard D. Wright, Endrews Delbaje, Bradley Laflamme, Sara Fallah, Evelyne Côté, Nicole Robbins, Thaila Fernanda dos Reis, Gustavo H. Goldman, Leah E. Cowen, Nir Osherov

**Affiliations:** 1 Department of Clinical Microbiology and Immunology, Gray School of Medicine, Tel Aviv University 26745, Tel Aviv, Israel; 2 David Braley Centre for Antibiotics Discovery, M.G. DeGroote Institute for Infectious Disease Research, Department of Biochemistry and Biomedical Sciences, McMaster University 3710, Hamilton, Ontario, Canada; 3 Departamento de Biologia, Faculdade de Filosofia, Ciências e Letras de Ribeirão Preto, Universidade de São Paulo 124589 , Ribeirão Preto, São Paulo, Brazil; 4 Department of Molecular Genetics, University of Toronto 204248, , Toronto, Ontario, Canada; 5 Departamento de Bioquímica, Instituto de Química, Universidade Estadual de São Paulo, Araraquara, Brazil; 6 Faculdade de Ciências Farmacêuticas de Ribeirão Preto, Universidade de São Paulo 67782 , Ribeirão Preto, São Paulo, Brazil; 7 National Institute of Science and Technology in Human Pathogenic Fungi, São Paulo, Brazil; University of Iowa, Iowa City, Iowa, USA

**Keywords:** *Aspergillus fumigatus*, triazole antifungals, drug resistance, evolution

## Abstract

*Aspergillus fumigatus* is the leading cause of invasive mold infections in immunocompromised patients. Current antifungal treatment primarily depends on the triazole antifungals, which act by inhibiting Erg11/Cyp51, a key enzyme in the ergosterol biosynthetic pathway. However, resistance is emerging at an increasing rate, reducing treatment efficacy and patient survival. Confirmed resistance mechanisms in clinical isolates include mutations in *cyp51A*, *cyp51B*, *hmg1*, *hapE*, *cox10*, and the overexpression of drug efflux pumps. To identify additional determinants of triazole resistance, we grew *A. fumigatus* wild-type and *Δcyp51A* mutant strains under increasing concentrations of voriconazole. Sequencing of the resultant resistant strains identified known mutations in *cyp51A* and *cyp51B*, and novel mutations in *hmg1*, *abcC (cdr1B)*, *ptaB, erg25B,* and *srbA*. Mutations of *hmg1* and *ptaB* occurred early during evolution, while mutations of *erg25B* and *srbA* occurred later. Reintroduction of the novel mutations in *hmg1*, *abcC*, *ptaB,* and *erg25B* into wild-type *A. fumigatus* and correction of the *srbA* mutation in the evolved strain validated their contribution toward triazole resistance. Sterol profiling analysis indicated that mutation or deletion of *erg25B* is associated with a decrease in the accumulation of methylated sterols. Mutation or deletion of *ptaB* resulted in increased *cyp51A*, *cyp51B*, and *erg25A* expression. Sequence analysis of clinical isolates revealed enrichment of missense mutations in *ptaB*, *hmg1*, *abcC*, and *srbA* among triazole-resistant strains.

## INTRODUCTION

Systemic or invasive fungal infections represent a growing and significant public health concern, with an estimated 6.5 million cases and approximately 2.5 million associated deaths annually. The most prevalent fungal pathogens include *Candida* species, *Aspergillus* species, and *Cryptococcus neoformans* (1).

*Aspergillus fumigatus* is the most common invasive mold pathogen in humans, although other species such as *Aspergillus flavus*, *Aspergillus niger*, and *Aspergillus terreus* are also known to cause disease (2). *A. fumigatus* can cause a wide range of diseases, including allergic bronchopulmonary aspergillosis, chronic pulmonary aspergillosis, fungal sinusitis, and invasive pulmonary aspergillosis (IPA) (3, 4). It is estimated that there are approximately 3 million cases of chronic pulmonary aspergillosis and over 2 million cases of IPA annually worldwide, with treated mortality rates ranging from 40% to 60% (1).

Antifungal treatment options are limited because fungi are eukaryotes, sharing many cellular structures with humans, making selective targeting of fungal cells challenging. Only a limited number of drugs are effective against IPA, including the relatively toxic amphotericin B, echinocandins, and triazoles (5). The first-line treatment for diagnosed IPA is the triazole antifungals, which inhibit Erg11/Cyp51 sterol demethylase activity, thereby blocking ergosterol biosynthesis and leading to the accumulation of toxic 14α-methylated sterols (6, 7). Ergosterol is an essential component of fungal membranes, and its depletion results in membrane instability, increased permeability, and ultimately fungal cell death. However, the widespread use of azole fungicides in agriculture and prolonged antifungal therapy in patients with chronic or invasive aspergillosis have contributed to the emergence of triazole-resistant *A. fumigatus* strains, leading to increased mortality (6, 8, 9). The prevalence of triazole resistance in *A. fumigatus* varies widely across geographic regions, clinical centers, and patient populations, ranging from 1.7% to 28%, with higher rates generally reported in Northern Europe (10–13).

To date, the most commonly reported triazole resistance mechanism in *A. fumigatus* isolates worldwide involves mutations in the *erg11A/cyp51A* gene and insertion of a tandem repeat (TR) in the promoter region (TR34/L98H or TR46/Y121F/T289A). The TR introduces two additional binding sites for the positive transcriptional regulator SrbA, leading to overexpression of the mutated Cyp51A enzyme. Concurrently, the amino acid substitutions within the coding region reduce the binding affinity of triazoles to their target (14). These resistance mutations are believed to have emerged in environmental *A. fumigatus* strains following prolonged exposure to high concentrations of agricultural azole fungicides (8). Additional *cyp51A* mutations associated with triazole resistance have been identified in patients undergoing long-term antifungal therapy. The most frequently observed substitutions occur in the Cyp51A ligand access channel (e.g., G54, P216, F219, and M220) or near the catalytic site (e.g., Y121 and G138) (15). Notably, only approximately half of all sequenced triazole-resistant clinical isolates harbor a resistant allele of *cyp51A,* indicating that alternative resistance mechanisms are also involved (16, 17).

Additional mechanisms contributing to clinical triazole resistance in *A. fumigatus* include a G457S substitution in *cyp51B* (18, 19), overexpression of the ABC transporter *abcC* (20, 21), and mutations in *hmg1*, encoding HMG-CoA reductase, the enzyme catalyzing the first committed step in ergosterol biosynthesis (21–25). Other reported mechanisms contributing toward clinical resistance include a P88L substitution in *hapE,* a transcriptional regulator that forms part of a *cyp51A* repressor complex (26, 27), an R243Q substitution in *cox10*, a mitochondrial gene involved in heme biosynthesis (28), and an E180D substitution in the 29.9 kDa subunit of mitochondrial complex I (29).

While numerous mechanisms contributing to triazole resistance have been identified in clinical and environmental *A. fumigatus* isolates, a substantial proportion of resistant strains lack known mutations, suggesting the existence of additional, as-yet-undiscovered mechanisms of resistance. To explore these, we employed experimental evolution as a tool to uncover novel genetic determinants of resistance, given that this is a powerful approach for uncovering novel fungal resistance mechanisms (30, 31). In this study, we employed a stepwise evolution approach by exposing *A. fumigatus* strains to gradually increasing concentrations of voriconazole (VRC). The resulting resistant isolates were subjected to whole-genome sequencing, leading to the identification of novel mutations. These mutations were subsequently verified and investigated for their mechanistic roles in resistance.

## MATERIALS AND METHODS

### Media and strains

Strains were grown on YAG agar plates (0.5% yeast extract, 1% dextrose, 0.01 M MgSO_4_, trace elements solution, vitamin mix, and 1.5% agar) for 48–72 h at 37°C. Tween 20 solution (0.02%) was used to collect the conidia. Microdilution or minimal inhibitory concentration (MIC) experiments were performed in RPMI-MOPS broth (10% [vol/vol] RPMI 10× and 3.45% [wt/vol] MOPS, pH 7.0), while droplet assays were performed on YAG agar plates. Mycelium was prepared for transformation by static incubation in 15 mL SAB broth in petri dishes for 16–20 h at 37°C. *A. fumigatus*-transformed protoplasts were plated on YPGS agar plates for hygromycin (Hyg) selection (2% yeast extract, 0.5% peptone, 2% D-glucose, 1 M sucrose, 1.5% agar for plates or 0.7% for top, and 250 μg/mL Hyg, pH 6) or AMM sucrose agar plates for pyrithiamine selection (1× salts solution, 1% dextrose, 1 M sucrose, 0.012 M KPO_4_, pH 6.8, 0.1% trace elements solution, 1.5% agar for plates or 0.7% for top, and 0.1 μg/mL pyrithiamine, pH 6). Transformation solutions include TRAFO 1 (0.6 M KCl, 50 mM CaCl_2_, 5 mM Tris-HCl, pH 7.5, autoclaved), TRAFO 2 (40% PEG3350 in TRAFO 1, autoclaved), digestion solution (10 mL TRAFO1 and 5% VinoTaste FCE [Novo Nordisk], filter-sterilized), and Cas9 Working buffer (20 mM HEPES and 150 mM KCl, pH 7.5, filter-sterilized). The mutant strains described in this report were generated using the akuB^KU80-^ non-homologous end joining mutant derived from patient isolate CEA10 (32). A detailed description of their construction and verification can be found in the Supplemental material and Table S1. All strains were stored in 25% glycerol solution at −80°C. pTel-hyg^R^ was a generous gift from the laboratory of Prof. Amir Sharon at Tel Aviv University.

### Experimental evolution of VRC resistance

Experimental evolution was performed to generate *A. fumigatus* strains with increased VRC resistance. Four independent lineages were established from both the parental akuB^KU80-^ wild-type (WT) and *Δcyp51A* mutant backgrounds. Each lineage was serially passaged on YAG agar containing gradually increasing VRC concentrations, starting at 0.25 µg/mL for the wild-type and 0.125 µg/mL for the *Δcyp51A* strain, with 0.25–1 µg/mL increments at each passage. At each round, conidia from the entire plate were harvested in 0.02% Tween 20, and ~1 × 10^⁶^ conidia were plated onto fresh YAG + VRC plates at the next concentration. Control lineages were passaged in parallel on drug-free YAG. Evolution continued until strains ceased conidiating or exhibited complete growth inhibition. The total number of passages ranged from 12 to 30. Final populations were purified by two streaks on drug-free YAG and stored in 25% glycerol at −80°C. To monitor mutation emergence, conidia from each generation were preserved at −80°C, genomic DNA was extracted, and mutations were tracked by the amplification refractory mutation system (ARMS)-PCR and Sanger sequencing.

### Whole-genome sequencing and analysis

Genomic DNA from all final evolution-derived isolates was extracted and verified using agarose gel electrophoresis. DNA concentration and purity were measured with a NanoDrop spectrophotometer and a Qubit RNA/DNA HS Assay Kit (Thermo Fisher). Libraries were prepared using the NEBNext Ultra II FS DNA Library Prep Kit for Illumina (NEB), and their quality was assessed with a TapeStation system. The prepared libraries were then loaded onto the NextSeq 500/550 Mid Output Kit version 2.5 (300 cycles) (Illumina) and sequenced using the NextSeq 500 platform. Data analysis was conducted at the Core Facility of the Weizmann Institute (Rehovot, Israel).

### Fitness tests

Freshly collected conidia were counted and diluted as needed. For the sporulation tests, 10^3^ (YAG) or 10^4^ (YAG + 1/16 MIC VRC) conidia were plated on 25 mL agar plates in triplicate and incubated for 48 h at 37°C. A volume of 3 mL of 0.02% Tween 20 was used to collect as many conidia as possible from plates, and conidial stocks were diluted and counted. For the radial growth tests, 10^3^ conidia in a 10 μL drop were point inoculated on agar plates in triplicate and incubated for 48 h at 37°C (10 mL plates).

### Antifungal susceptibility testing

MIC assays were performed using CLSI M38-A2 broth microdilution methodology. Briefly, VRC, itraconazole (ITC), posaconazole (POS), isavuconazole (ISV), amphotericin B (AMB), or caspofungin (CAS) were diluted in RPMI-MOPS and loaded into 96-well plates. Conidia were diluted to 5 × 10^4^ conidia/mL and loaded into the wells. Plates were incubated at 37°C for 48 h, then the lowest concentration of antifungal in which no fungal growth was seen (observed by inverted light microscope) was set as the MIC. For CAS, the lowest concentration of antifungal in which fungal growth was severely impaired (observed by inverted light microscope) was set as the minimal effective concentration (MEC). Droplet susceptibility assays were performed by inoculation of 10^4^, 10^3^, 10^2^, or 10 conidia in 10 µL 0.02% Tween 20 drop on the surface of YAG agar plates containing different concentrations of VRC and incubated for 48 h at 37°C. Unless otherwise stated, all chemicals were purchased from Sigma-Aldrich (St. Louis, MI, USA).

### Construction of *A. fumigatus* repair templates

The target genes were amplified from the VRC-evolved strains in which these mutations were found. PCR was performed with Q5 High-Fidelity 2× Master Mix (NEB) using the primers detailed in Table S3. The entire PCR reaction was run on a 1% agarose gel, and the desired band was excised and cleaned with the Wizard SV Gel and PCR Clean-Up kit (Promega). DNA concentration was assessed using Nanodrop (Thermo Fisher).

### crRNA design, RNA duplex, and Cas9

Two crRNAs were designed for each gene (Table S4), targeting the 5′ and 3′ areas of the gene, using EuPaGDT: a web tool tailored to design CRISPR guide RNAs for eukaryotic pathogens (33). crRNAs, tracrRNA, and Cas9 nuclease were ordered from Integrated DNA Technologies (Iowa, USA).

### Protoplasting and transformation of *A. fumigatus*

The protoplasting and transformation protocol used to generate mutations or deletions in *erg25B*, *srbA,* and *ptaB* was based on the CRISPR-Cas9 protocol described in reference 34 (see the Supplemental material for details). The transformation protocol used to seamlessly generate the mutations in *hmg1* and *abcC* was adapted from the TRAFO-based protocol (35) and CRISPR-Cas9 protocol (34) (see the Supplemental material for details). This protocol uses the recyclable linear plasmid pTel-hyg^R^, which is described in detail in reference 36. Briefly, seamless genome editing in *A. fumigatus* was performed by *in vitro*-assembled CRISPR-Cas9 ribonucleoprotein complexes together with a PCR-amplified repair template carrying the desired mutation. Two guide RNAs targeting the 5′ and 3′ regions of the gene directed Cas9 cleavage, enabling precise replacement of the endogenous locus by homologous recombination using short flanking regions. A linear pTel-hygR plasmid carrying telomeric ends was co-transformed as a recyclable selectable marker to enrich for successfully transformed protoplasts. Three days post-transformation, ~3 mL of 0.02% Tween 20 was used to collect all conidia generated on the transformation plates; usually, conidia from three identical transformation plates were pooled. Conidia (10^3^–10^5^) from the transformation plates were seeded on YAG agar plates supplemented with 0.25–2 μg/mL VRC and incubated for 3–5 days at 37°C. This was done for the pTel-hyg^R^ + repair template conidia and for the pTel-hyg^R^-transformed control conidia. After incubation, four to six VRC-resistant colonies from pTel-hyg^R^ + repair template and one VRC-susceptible colony from pTel-hyg^R^ were streaked twice on fresh YAG agar plates and archived. After the two streaks on YAG agar plates, the final isolates were tested on YAG supplemented with 350 μg/mL Hyg for verification of pTel-hyg^R^ plasmid loss.

### Rapid genomic DNA extraction, ARMS-PCR, and sequence verification

From each isolate plate, a loop dipped in sterile double-distilled water (DDW) was used to collect conidia and then dipped into an Eppendorf tube containing 500 μL of DDW, until the liquid turned murky green. The tubes were frozen in liquid nitrogen for 15 min, then immediately transferred to 100°C for 5 min. Tubes were vortexed vigorously for 15 s and spun down to pellet cell debris. The upper, clear, aqueous phase was taken for further use. PCR reactions were performed using Red Taq 5× Master Mix (Larova). Screening of the isolates for the presence of the desired mutations was done by amplification refractory mutation system PCR (37). ARMS-PCR primers, listed in Table S5, were designed using the University of Southampton PRIMER1: primer design for tetra-primer ARMS-PCR website. The final mutant isolates from each transformation were verified for the presence of the mutation through Sanger sequencing.

### Extraction and mass spectrometry analysis of sterols

To prepare samples for sterol profiling, 1 × 10^4^ conidia/mL of each *A. fumigatus* strain was added to two independent 250 mL flasks containing 50 mL YPD (for a total of six flasks) and incubated for 20 h at 37°C and 150 rpm. After 20 h, flasks for each strain were treated with either 2 µg/mL VRC or an equivalent volume of DMSO and returned to incubate at 37°C and 150 rpm for 4 h. At the 24 h time point, *A. fumigatus* pellets were collected via vacuum filtration, washed with cold 1× PBS, flash frozen in liquid nitrogen, and stored at −80°C until harvesting of sterols.

For sterol extraction, pellets were crushed using a mortar and pestle cooled with liquid nitrogen. Pellets were then weighed and resuspended in 3 mL extraction solution (25% KOH [wt/vol] and 64% methanol [vol/vol]) and transferred into borosilicate glass tubes. A volume of 30 µL of 2 mg/mL cholesterol (dissolved in chloroform) was added as an internal standard, and samples were incubated in an 80°C water bath for 1 h and vortexed every 20 min. After incubation, samples were cooled to room temperature, and 1 mL of water and 3 mL of chloroform were added to each sample for extraction. The tubes were then vortexed for 10 s three times and then centrifuged at 1,048 × *g* for 5 min. Using Pasteur pipettes, the bottom organic chloroform phases were transferred to 20 mL liquid scintillation and dried by Genevac (EZ-2 Series SP Scientific) on the very low boiling point setting.

For GC-MS derivatization, samples were dissolved in 600 µL chloroform, and 200 µL was then transferred into 1 mL conical thick-walled glass reaction vials for derivatization, as previously reported. The bottom of the tubes was suspended in a 35°C water bath, and lipid extracts were evaporated under a stream of N_2_ gas at an airflow rate of 1 mL/min. The extract was dissolved in 20 µL pyridine and 30 µL MSTFA by flicking the bottom of the vial. Next, samples were incubated in a dry heating block at 37°C for 30 min to derivatize. After derivatization, the samples were centrifuged at 13,000 rpm for 10 min. Then, 20 µL of the supernatant was transferred into GC auto-sampler vials, and 1 µL of the extract was injected into the GC-MS for analysis. Each sample was analyzed in duplicate. Sterols were analyzed using an Agilent GC/MS system (HP7890/HP5975) equipped with a DB-5MS column (Agilent 122-5532; 30 m × 0.25 mm ID, 0.25 μm film thickness). Samples were injected at 250°C into a heated inlet, with helium as the carrier gas at a constant flow rate of 1 mL/min. The oven temperature was isocratic at 50°C for 2.5 min, then increased to 70°C at a rate of 7.5°C/min, followed by an increase in temperature to 320°C at a rate of 10°C/min and held for 5 min. The oven was then cooled to 50°C before injecting the next sample. The MS detector was turned on at 6 min, and the mass spectra were recorded at 2.3 scans/s, with a scanning range of 50–700 *m/z*. Peak areas of sterols were normalized to that of internal standards and the wet weights of each sample. Medians between the two technical replicates of the three biological replicates of each sample were plotted as a bar graph with individual data points using Prism version 10.2.2.

### Determination of gene expression by qPCR

Strains were grown on YAG agar plates for 72 h at 37°C; 10^7^ conidia were loaded into six flasks containing 25 mL YAG each. The flasks were incubated with shaking at 150 rpm for 20 h at 37°C. Then, a 0.5 MIC concentration of VRC (Tables 1 to 3) was added to three flasks, and three flasks were left untreated. Flasks were incubated with shaking at 150 rpm for 4 h at 37°C. Mycelium was collected, lyophilized, and crushed. RNA was extracted using the QIAGEN RNeasy Plant Mini Kit. The concentration was assessed using NanoDrop, then equal amounts of RNA from each sample were converted to cDNA using the Verso cDNA Synthesis Kit (Thermo Fisher). Equal volumes of cDNA from each sample were loaded into Applied Biosystems MicroAmp Optical 96-Well plates with Applied Biosystems Fast SYBR Green Master Mix and primers for either β-tubulin (housekeeping control gene) or the other genes that were tested (Table S6). 2^-ΔΔCt^ analysis was performed. Statistical analysis was performed with one-way ANOVA, unless stated otherwise.

**TABLE 1:** Mutations and antifungal susceptibility (MIC) profiles of voriconazole-evolved *A. fumigatus* isolates^*a*^^,^*^b^*^,^*^d^*

Isolate	Genotype	MIC (μg/mL)					
		VRC	ITC	POS	ISV	AMB	CAS
*ΔKU80* (WT):							
Control 29	*Δku80*	0.5	0.5	0.06	1	0.5	0.25
A20	*Δku80; cyp51A* G448S; AFUB_017180 T850I, I851T; AFUB_031920/ras GEF 1067frameshift; AFUB_101520/histidine kinase G174frameshift; AFUB_101620 T61A	16	>16	0.5	>8	0.5	0.25
B29	*Δku80; cyp51B* G457S; ***abcC* R339H; *erg25B* D253G**; AFUB_017180 T850I, I851T; AFUB*_*073840/*creC* L326S; AFUB_047570 P1240S; AFUB_056670 S203frameshift; AFUB_071010 R927K, S926F, T920M, R827W	>16	>16	1	>8	1	0.125
C12	*Δku80;* ***erg25B* G178D; *ptaB* Q397frameshift**; AFUB_047540/*pyrG* E77T; AFUB_021830 E829stop; AFUB_066290 L167P	8	>16	1	>8	0.5	0.25
D12	*Δku80;* ***erg25B* G178D; *ptaB* Q397frameshift**; AFUB_047540/*pyrG* T10S, A21V, R23K, I27V, L50F, E77T, A192H; AFUB_066290 L167P; AFUB_006290 T350A; AFUB_021830 E829stop	8	>16	1	>8	0.5	0.25
*Δcyp51A* background							
Control 30	*Δku80; Δcyp51A*/*hph*	0.125	0.03	0.016	0.25	0.5	0.25
A3	*Δku80; Δcyp51A*/*hph*; AFUB_047540 A192H; AFUB_079180 3^*c*^; AFUB_079190 5^*c*^; AFUB_079200 V8I; AFUB_079210 2^*c*^; AFUB_079220 16^*c*^; AFUB_079230 21^*c*^; AFUB_079240 17^*c*^; AFUB_079250 4^*c*^; AFUB_079260 4^*c*^; AFUB_079300 S1765F; AFUB_079320 2^*c*^; AFUB_079330 7^*c*^; AFUB_079350 2^*c*^; AFUB_079360 2^*c*^; AFUB_079380 4^*c*^; AFUB_079420 H187Y; AFUB_079430 V190M; AFUB_079450 V37A; AFUB_079460 V37A; AFUB_079470 2^*c*^; AFUB_079470 2^*c*^; AFUB_079470 5^*c*^; AFUB_079520 6^*c*^; AFUB_079540 R517P; AFUB_079610 G254A; AFUB_079610 F91L; AFUB_079650 3^*c*^; AFUB_079660 E218Q; AFUB_079670 2^*c*^; AFUB_079670 2^*c*^; AFUB_079870 3^*c*^; AFUB_101520 G174frameshift; AFUB_102200 V190I	1	0.25	0.25	2	0.5	0.125
B12	*Δku80; Δcyp51A*/*hph*; ***hmg1* G386W; *abcC* S570N; *ptaB* Q312stop; *srbA* L250P**; AFUB_047540/*pyrG* A192H	8	0.5	0.06	8	0.5	0.25
C30	*Δku80; Δcyp51A*/hph; *cyp51B* G457S; *hmg1* F262del**; *erg25B* K294frameshift**; AFUB_047540/*pyrG* A192H; AFUB_025800 N98D/h*sp70*; AFUB_010840 K200/stop/*ubcE2*	>16	1	1	>8	0.5	0.25
D2	*Δku80; Δcyp51A*/*hph*; ***hmg1* L493P**	1	0.25	0.25	2	0.5	0.25

^*a*^ Underline, known mutations found in key genes.

^*b*^ Bold, novel mutations found in key genes.

^*c*^ Number of mutations found in the gene.

^*d*^ *cyp51A,* AFUB_063960; *cyp51*B, AFUB_089270; *hmg1,* AFUB_020770;* abcC,* AFUB_013880;* erg25B,* AFUB_098170; *ptaB, * AFUB_028530; and* srbA,* AFUB_018340.

**TABLE 2:** Main mutations found in evolution-derived isolates

	Cyp51A	Cyp51B	Hmg1	SrbA	Erg25B	AbcC	PtaB
*ΔKU80*	G448S	G457S			G178D D253G	R339H	Q397 frameshift
*Δcyp51A*		G457S	F262del G386W L493P	L250P	K294 frameshift	S570N	Q312stop

**TABLE 3:** MICs of reconstituted mutations (WT background)

	MIC (μg/mL)					
	VRC	ITC	POS	ISV	AMB	CAS
*ΔKU80* (WT)	0.5	0.25	0.015	1	0.5	0.25
Cyp51A G448S	4	>16	0.25	8	0.5	0.25
Cyp51B G457S	2	0.5	0.03	4	0.5	0.25
Hmg1 F262del	4	>16	0.25	8	0.5	0.25
Hmg1 G386W	1	>16	0.125	4	0.5	0.25
Hmg1 L493P	2	>16	0.25	4	0.5	0.25
AbcC R339H	1	0.25	0.015	1	0.5	0.25
Erg25B D253G-PtrA	1	1	0.06	2	1	0.25
*Δerg25B*	1	0.5	0.03	2	0.5	0.25
PtaB Q312stop	2	>1^*a*^	0.06	4	0.5	0.06
PtaB Q397frameshift-PtrA	2	>1^*a*^	0.06	4	0.5	0.06
*ΔptaB*	2	>1^*a*^	0.06	4	0.5	0.06
SrbA L250P-hph	0.25	0.06	0.015	0.25	1	0.25
*ΔsrbA*	0.125	0.015	0.008	0.06	1	0.25

^*a*^ At ITC concentrations higher than 1 μg/mL, precipitation crystals form, leading to decreased inhibition.

### Determination of the timeline of mutation emergence

Conidia from all generations of each lineage were preserved at –80°C in 0.02% Tween 20 and 25% glycerol. For each generation, conidia were plated on fresh YAG agar, followed by rapid genomic DNA extraction and ARMS-PCR, as previously described. The emergence of a given mutation was inferred from the gradual appearance and intensification of the mutant-specific ARMS-PCR band, accompanied by a corresponding reduction in the WT band. Generations within this transition window were subjected to Sanger sequencing. The relative abundance of the WT and mutant alleles at the specific nucleotide position was assessed, and the mutation was defined as having occurred in the generation where the mutant allele reached at least 10% of the population.

### Identification of genetic variants and mutation analysis from whole-genome sequencing data

Reads from publicly available WGS projects were processed for quality control. The quality of the sequences was assessed using FastQC version 0.11.8 (38), and the adapter sequences were trimmed and filtered by quality using Trimmomatic version 0.39 (39), with the parameters “ILLUMINACLIP:NexteraPE-PE.fa:2:30:10 LEADING:20 TRAILING:20 MINLEN:60.” To detect the variants, the reads were first mapped to the reference genome of *A. fumigatus* Af293 (NCBI assembly code GCA_000002655.1) using Bowtie version 2.3.5.1 (40), and the generated SAM files were converted to BAM format with SAMtools version 1.15.1 (41). Single nucleotide polymorphisms (SNPs) were identified using the Genome Analysis Toolkit version 4.3.0.0 (42) with the module “HaplotypeCaller.” Low-confidence SNPs were filtered using “VariantFiltration” with the parameters “QualByDepth < 25.0 || MappingQuality < 55.0 || MappingQualityRankSumTest < −0.5 || FisherStrand > 5.0 || StrandOddsRatio > 2.5 || ReadPosRankSumTest < −2.0.” The VCF files were annotated with SnpEff using the genome Af293 as reference (GCA_000002655.1) (43).

Genetic variants identified were assessed for their association with phenotypic groups (susceptible, *cyp51A*-dependent resistance, and *cyp51A*-independent resistance). Association analysis was performed by calculating odds ratios and corresponding *P*-values using the oddsratio function from the epitools package in R (44). The statistical significance was determined using Fisher’s Exact Test.

## RESULTS

### Stepwise evolution of *A. fumigatus* on gradually increasing levels of VRC generates resistant strains

**Fig 1:**
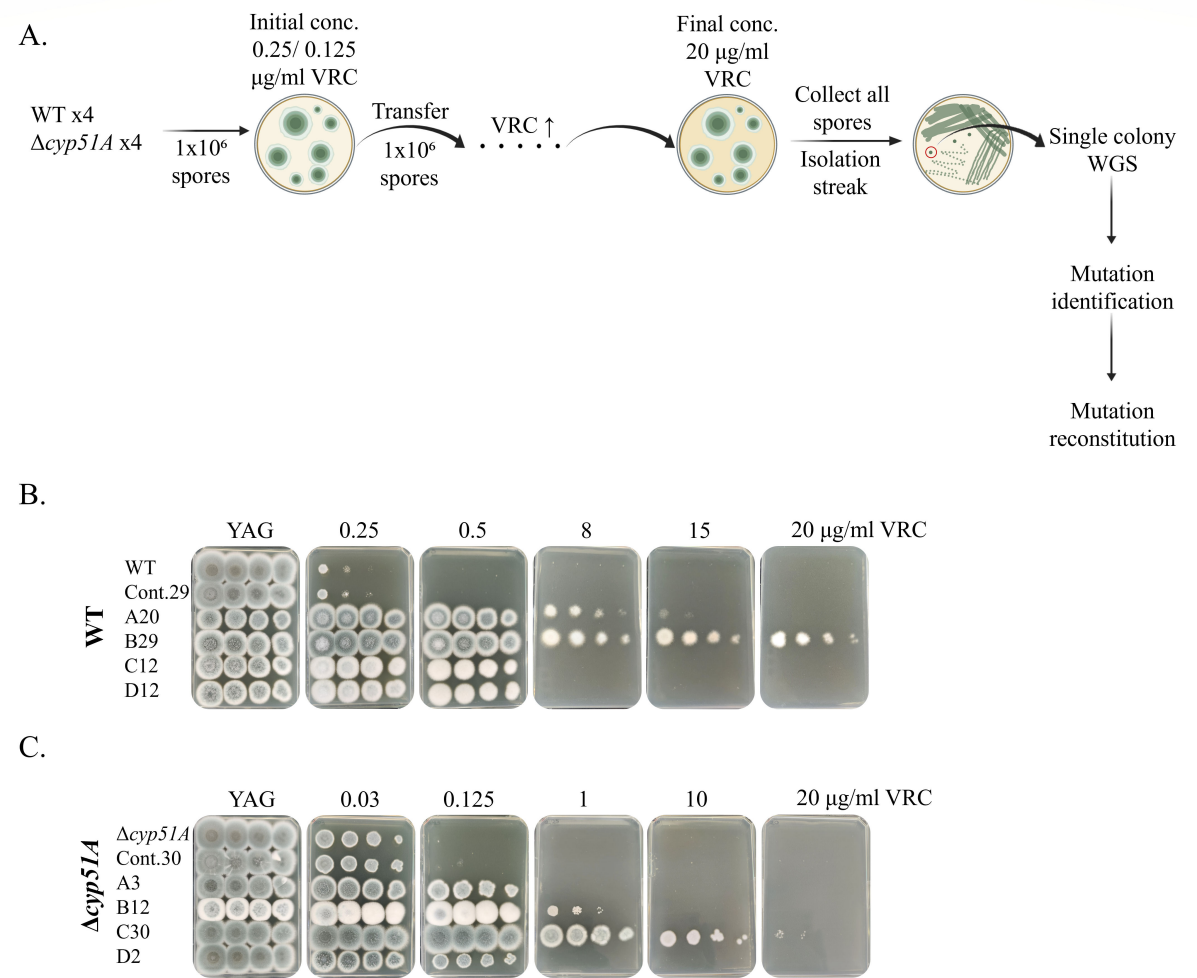
Stepwise evolution of *A. fumigatus* under increasing VRC concentrations generates resistant strains. (**A**) Schematic representation of the stepwise evolution protocol. (**B**) Serial dilution assays of independently evolved VRC-resistant strains (A20, B29, C12, and D12) derived from the *akuB^KU80-^* wild-type background under increasing VRC concentrations. The numeral denotes the total number of passages. Control strain Cont.29 represents *akuB^KU80-^* passaged 29 times without drug. (**C**) Serial dilution assays of independently evolved VRC-resistant strains (A3, B12, C30, and D2) derived from the *Δcyp51A* background under increasing VRC concentrations. Control strain Cont.30 represents *Δcyp51A* passaged 30 times without drug.

**Fig 2:**
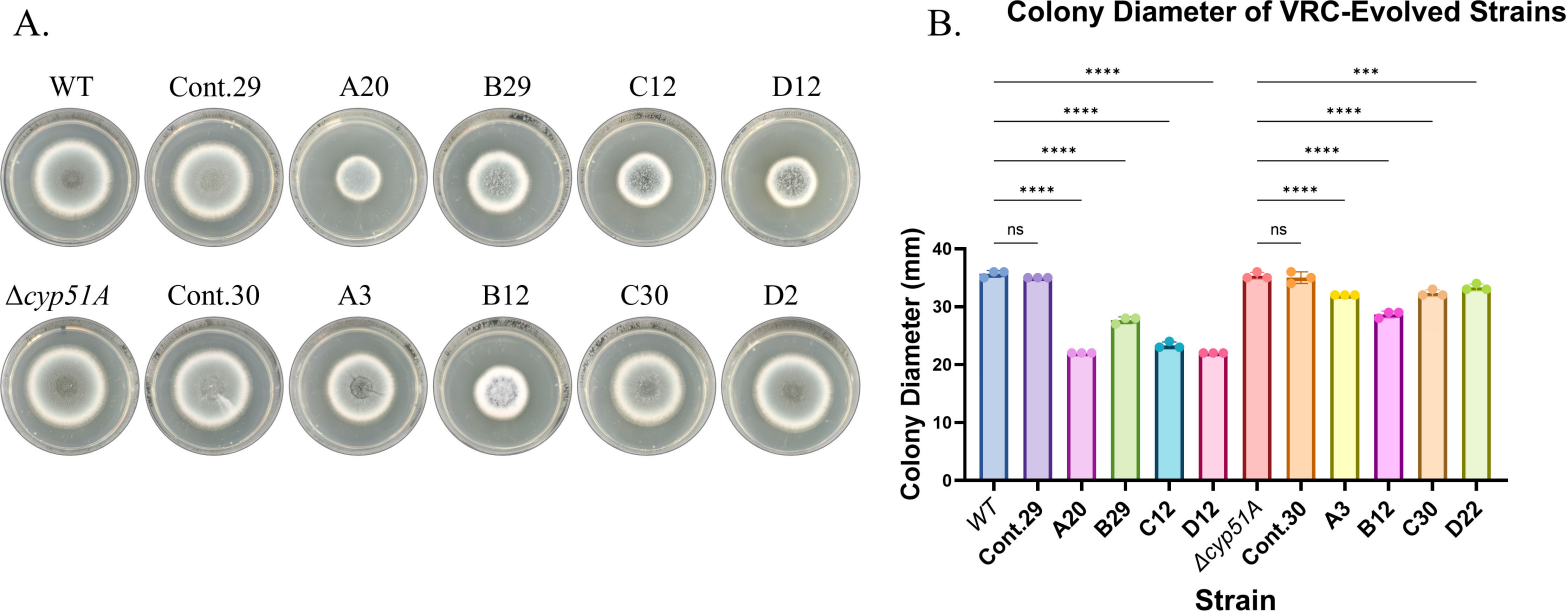
Evolved resistant strains exhibit a fitness cost in the absence of VRC. (**A**) Point inoculation of evolved and control strains on YAG agar, incubated for 72 h at 37°C. (**B**) Radial growth measurements showing significantly reduced colony diameter in evolved strains compared to controls (****, *P* < 0.0001). Error bars indicate the standard deviation of biological triplicate. ns, not significant.

To reveal novel mechanisms of triazole resistance in *A. fumigatus*, we performed stepwise evolution under increasing VRC concentrations. VRC was selected because it is the drug of choice for treating patients with invasive aspergillosis. Four plates of parental wild-type (WT/akuB^KU80-^) and four plates of *Δcyp51A* strains were serially passaged on YAG agar containing progressively higher VRC concentrations (Fig. 1). We hypothesized that subjecting the *Δcyp51A* strain, in addition to the WT, to evolutionary pressures may reveal resistance mechanisms that operate independently of the extensively characterized *cyp51A* mutations. Selection began at the subinhibitory concentrations of 0.25 µg/mL VRC for the WT strain and 0.125 µg/mL VRC for the VRC-sensitive *Δcyp51A* strain and increased by 0.25–1 µg/mL VRC at each step (Fig. 1A). Control WT (Cont.29) and *Δcyp51A* (Cont.30) strains were simultaneously passaged in the absence of VRC. The evolutionary process was terminated at 20 µg/mL or at the passage where the VRC concentration inhibited conidiation or growth entirely. Then, a single colony from the highest VRC concentration was selected for phenotypic and WGS analysis. Subsequent passaging of these isolated resistant strains without VRC demonstrated that they maintained stable resistance and displayed a consistent phenotype (data not shown). All WT isolates were verified to be *ΔKU80,* and all *Δcyp51A* background isolates were verified to be *Δcyp51A*, eliminating the possibility of contamination by other resistant *A. fumigatus* strains (data not shown). Strains A20, B29, C12, and D12 evolved from the WT strain (the letter denotes the strain lineage, and numbers denote the total number of passages it evolved) showed a 16- to >40-fold increase in VRC resistance on YAG agar (Fig. 1B) and an 8- to >32-fold increase in triazole (VRC/ITC/POS) resistance by standard broth microdilution assay (Table 1). Strains A3, B12, C30, and D2, evolved from the *Δcyp51A* strain, displayed an 8- to >128-fold increase in VRC resistance on YAG agar (Fig. 1C) and an 8- to >128-fold increase in triazole (VRC/ITC/POS) resistance compared to the *Δcyp51A* strain by standard broth microdilution assay (Table 1). In the absence of VRC, all resistant WT and *Δcyp51A*-evolved strains showed reduced radial growth (Fig. 2A and B), indicating that adaptation to elevated VRC levels is associated with a fitness cost in its absence.

### WGS of the evolved VRC-resistant *A. fumigatus* strains identifies mutations in *cyp51A, cyp51B, hmg1, abcC, erg25B, ptaB,* and *srbA*

To identify mutations contributing to VRC resistance, we performed WGS and sequence analysis of the evolved strains and compared them to the control strains passaged in parallel in the absence of VRC. The published sequence of the WT-CEA10 genome (NCBI accession number GCA_051225625.1) was used as a scaffold. Overall, the evolved VRC-resistant strains displayed limited coding mutations (between one and eight mutations), and a few single gene duplications and deletions (Table 1; Table S2). Strain A3 contained multiple mutations in a gene block spanning 31 genes and was not further studied (Table 1). We focused our analysis on coding mutations in genes that exhibited alterations across multiple, independently evolved strains, as we reasoned that they were most likely to contribute to resistance. Cyp51A G448S, Cyp51B G457S, and Hmg1 F262del substitutions were not further studied as they have been described previously (19, 36) (Table 1, underlined). Novel substitutions in Hmg1 (G386W and L493P), Erg25B (G178D, D253G, and K294FS), AbcC/Cdr1B (R339H), PtaB (Q397FS and Q312stop), and SrbA (L250P) were selected for further analysis (Table 1, bold, Table 2).

To assess the contributions of mutated *hmg1, abcC, erg25B, ptaB,* and *srbA* genes to triazole resistance, the mutated genes were introduced singly into the WT parental strain. Select genes were also introduced into the *Δcyp51A* parental strain. We used CRISPR-Cas9 to excise the WT version of the gene and replace it with the corresponding mutated version, as previously described (36). We generated three to four independent PCR-positive transformants for each strain, all of which exhibited a similar phenotype (see full details for strain construction and verification in the supplemental material). An additional control when introducing the mutated repair template juxtaposed to a selectable *hgh* or *ptrA* marker into *erg25B, ptaB,* and *srbA* was a parallel transformation with a non-mutated repair template (this control is shown in Fig. S3 to S5). To evaluate VRC sensitivity under the conditions used during evolution, all strains described below were point inoculated and grown for 2 days at 37°C on YAG agar plates containing increasing VRC concentrations. To determine triazole (VRC, ITC, POS, and ISA), CAS, and AMB sensitivity, we determined MICs by CLSI M38-A2 broth microdilution methodology (Tables 1, 3, and 4).

**TABLE 4:** MICs of reconstituted mutations (*Δcyp51A*)

	MIC (μg/mL)					
	VRC	ITC	POS	ISV	AMB	CAS
*Δcyp51A*-hph/*Δcyp51A*-PtrA	0.125	0.03	0.008	0.25	0.5	0.25
Hmg1 F262del	1	0.125	0.03	2	0.5	0.25
Hmg1 G386W	1	0.125	0.03	1	0.5	0.25
Hmg1 L493P	1	0.25	0.125	2	0.5	0.25
*Δcyp51A* B12 SrbA-fix	4	0.25	0.03	4	0.5	0.125

### Novel Hmg1 substitutions G386W and L493P contribute to triazole resistance in *A. fumigatus*

**Fig 3:**
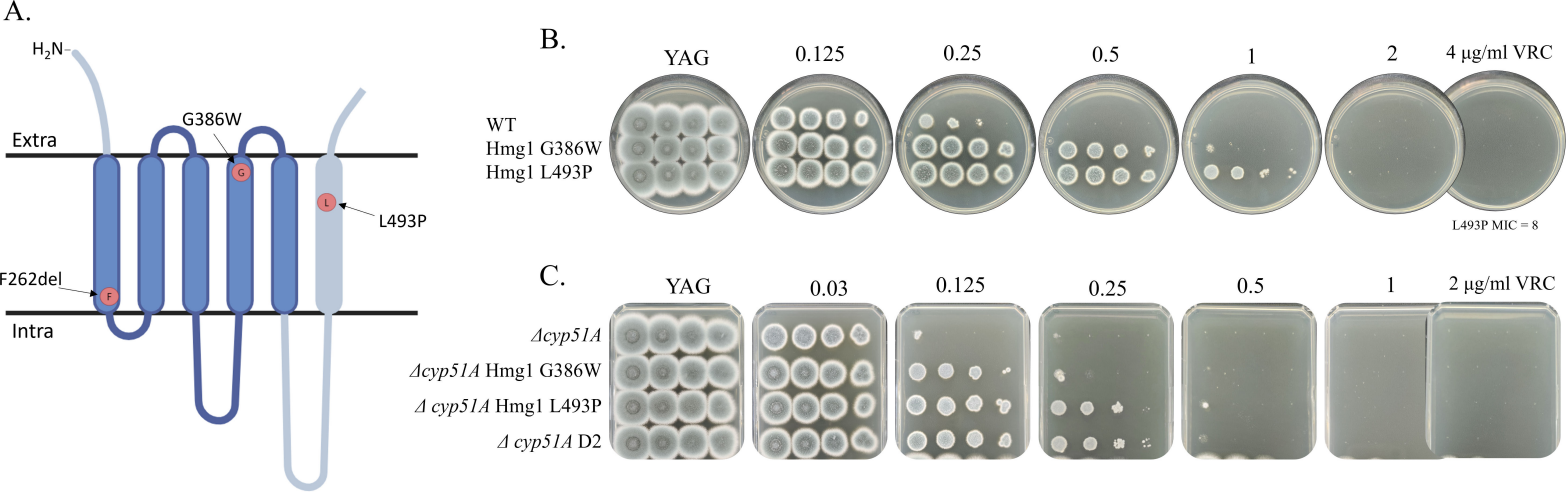
Novel Hmg1 substitutions G386W and L493P contribute to VRC resistance in *A. fumigatus*. (**A**) Schematic representation of Hmg1 showing the positions of G386W in the sterol-sensing domain and L493P outside the SSD. (**B**) Serial dilution assays of WT strains carrying Hmg1 G386W or L493P on YAG agar with increasing voriconazole concentrations. (**C**) Serial dilution assays of *Δcyp51A* strains carrying Hmg1 G386W or L493P on YAG agar with increasing VRC concentrations.

Hmg1 encodes HMG-CoA reductase, which catalyzes the first committed step in ergosterol biosynthesis. Recently, triazole resistance mutations in the sterol-sensing domain (SSD) of Hmg1 have been identified in *A. fumigatus* clinical isolates (21–24). In this study, we identified two novel Hmg1 substitutions, G386W and L493P, in strains B12 and D2 (*Δcyp51A* background). G386W is in the fourth transmembrane domain of the SSD, while surprisingly, L493P is in the sixth transmembrane domain outside the SSD (Fig. 3A). These non-synonymous mutations were introduced into *hmg1* in both the WT and *Δcyp51A* parental strains, generating Hmg1 G386W, Hmg1 L493P, *Δcyp51A* Hmg1 G386W, and *Δcyp51*A Hmg1 L493P strains, respectively. All strains showed a 4-fold increase in MICs to VRC on YAG agar plates (Fig. 3B and C) and a 2–16-fold increase in VRC/ITC/POS/ISV MICs by broth microdilution, with unchanged sensitivity to the non-azole antifungals CAS and AMB (Tables 3 and 4).

### Substitution R339H in AbcC contributes to weak triazole resistance

**Fig 4:**
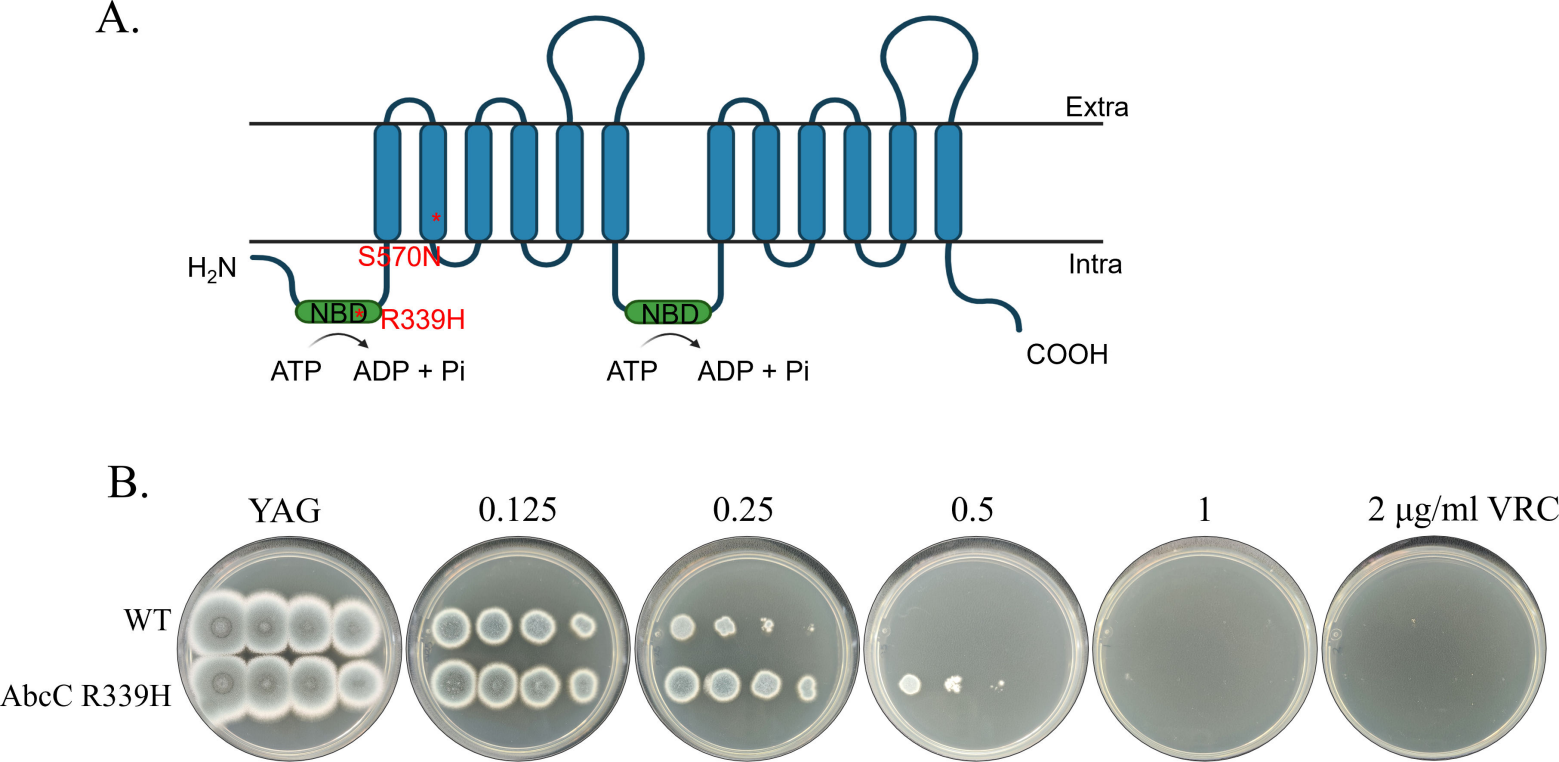
Novel AbcC substitution R339H contributes to VRC resistance in *A. fumigatus*. (**A**) Schematic representation of AbcC showing the positions of R339H in the nucleotide-binding domain (NBD) and S570N in the second transmembrane domain. (**B**) Serial dilution assays of WT strain carrying AbcC R339H on YAG agar with increasing VRC concentrations.

*AbcC* (alias *cdr1B* and *abcG1*) encodes an ATP-binding cassette efflux transporter that contributes to triazole resistance when overexpressed in *A. fumigatus* (20, 45). Here, we identified a novel *abcC* mutation, leading to a R339H substitution, within the first ATPase domain in strain B29 (WT background) (Fig. 4A), as well as a non-synonymous mutation, leading to a S570N substitution in strain B12 (*Δcyp51A* background). The R339H substitution was introduced into the WT parental strain, generating strain AbcC R339H. It showed a mild twofold increase in MICs to VRC on YAG agar plates (Fig. 4B) and a twofold increase in VRC MIC by broth microdilution (Table 3). However, AbcC R339H showed WT susceptibility to the triazoles ITC, POS, or ISV, or to CAS and AMB, indicating that this mutation confers a narrow window of weak resistance to VRC alone.

### Mutations in *erg25B* contribute to triazole resistance and are associated with a decrease in the accumulation of methylated sterols

**Fig 5:**
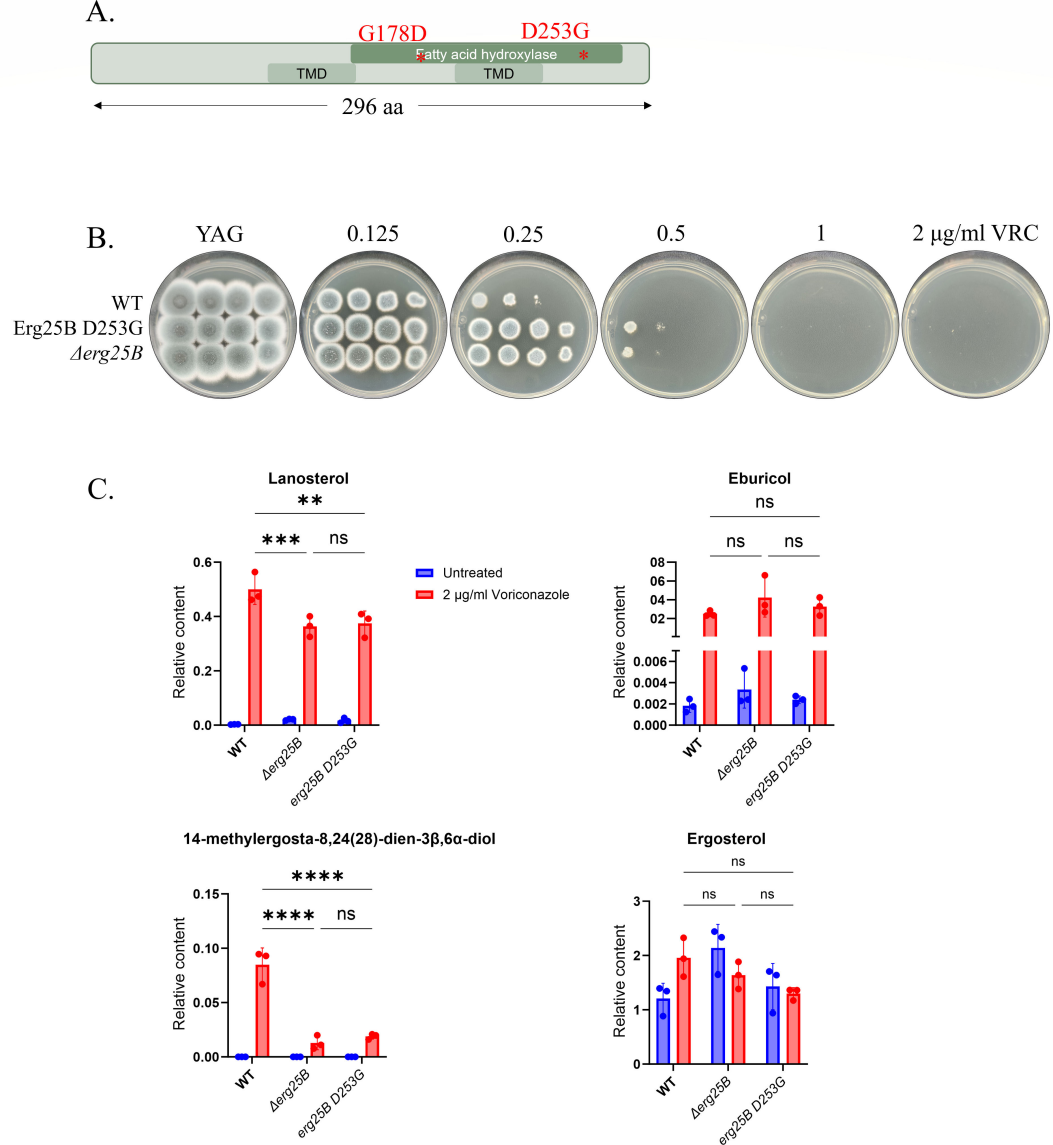
Novel Erg25B substitution D253G and *erg25B* deletion contribute to VRC resistance in *A. fumigatus*. (**A**) Schematic representation of Erg25B indicating the positions of G178D and D253G within the fatty acid hydroxylase domain. (**B**) Serial dilution assays of WT strain carrying Erg25B D253G or *erg25B* deletion on YAG agar with increasing VRC concentrations. (**C**) Sterol profiling of *akuB^KU80-^*, *Δerg25B*, and Erg25B D253G strains showing relative levels of lanosterol, eburicol, 14-methylergosta-8,24(28)-dien-3β,6α-diol, and ergosterol with or without VRC. Data are presented as mean ± SEM from three independent biological replicates, each performed in duplicate. ** *P* < 0.01, *** *P* < 0.001, **** *P* < 0.0001, ns, not significant.

*Erg25B* encodes a C4-sterol demethylase involved in generating fecosterol, an intermediate in the ergosterol biosynthesis pathway (46). We identified an Erg25B substitution (D253G) in the fatty acid hydroxylase domain in strain B29 and a G178D substitution in strains C12 and D12 (WT background). An Erg25B K294 FS (frameshift) mutation at the C-terminus of the protein was identified in strain C30 (*Δcyp51A* background). (Fig. 5A). Erg25B D253G was introduced into the WT parental strain, generating the strain Erg25B D253G. We also generated a strain in which *erg25B* was deleted (*Δerg25B*). Both strains showed a twofold increase in VRC MICs on YAG agar plates (Fig. 5B). By broth microdilution, Erg25B D253G and *Δerg25B* showed a two- to fourfold increase in VRC/ISV/ ITC/POS MICs (Table 3).

Sterol profiling was performed on WT, Erg25B D253G, and *Δerg25B* strains grown in YPD liquid medium for 20 h, followed by an additional 4 h with or without 2 µg/mL VRC. As expected, VRC treatment led to a significant increase in lanosterol and eburicol levels in all three strains (Fig. 5C). However, there were no significant differences in these sterol levels between the strains. Notably, ergosterol levels were not significantly reduced in any of the VRC-treated strains, likely due to the relatively short (4 h) drug exposure.

We observed an accumulation of 14-methylergosta-8,24(28)-dien-3β,6α-diol, a toxic methylated sterol, in the WT strain. Interestingly, VRC treatment resulted in no significant accumulation of this diol in the *Δerg25B* mutant and only minimal accumulation in the Erg25B D253G strain, relative to WT (Fig. 5C). These findings suggest a possible mechanism of decreased susceptibility to VRC in the *Δerg25B* and Erg25B D253G strains, through the reduced accumulation of toxic methylated sterols that are otherwise produced during antifungal treatment (see Discussion).

### Mutations in *ptaB* contribute to triazole resistance and increase the expression of key ergosterol biosynthesis genes

**Fig 6:**
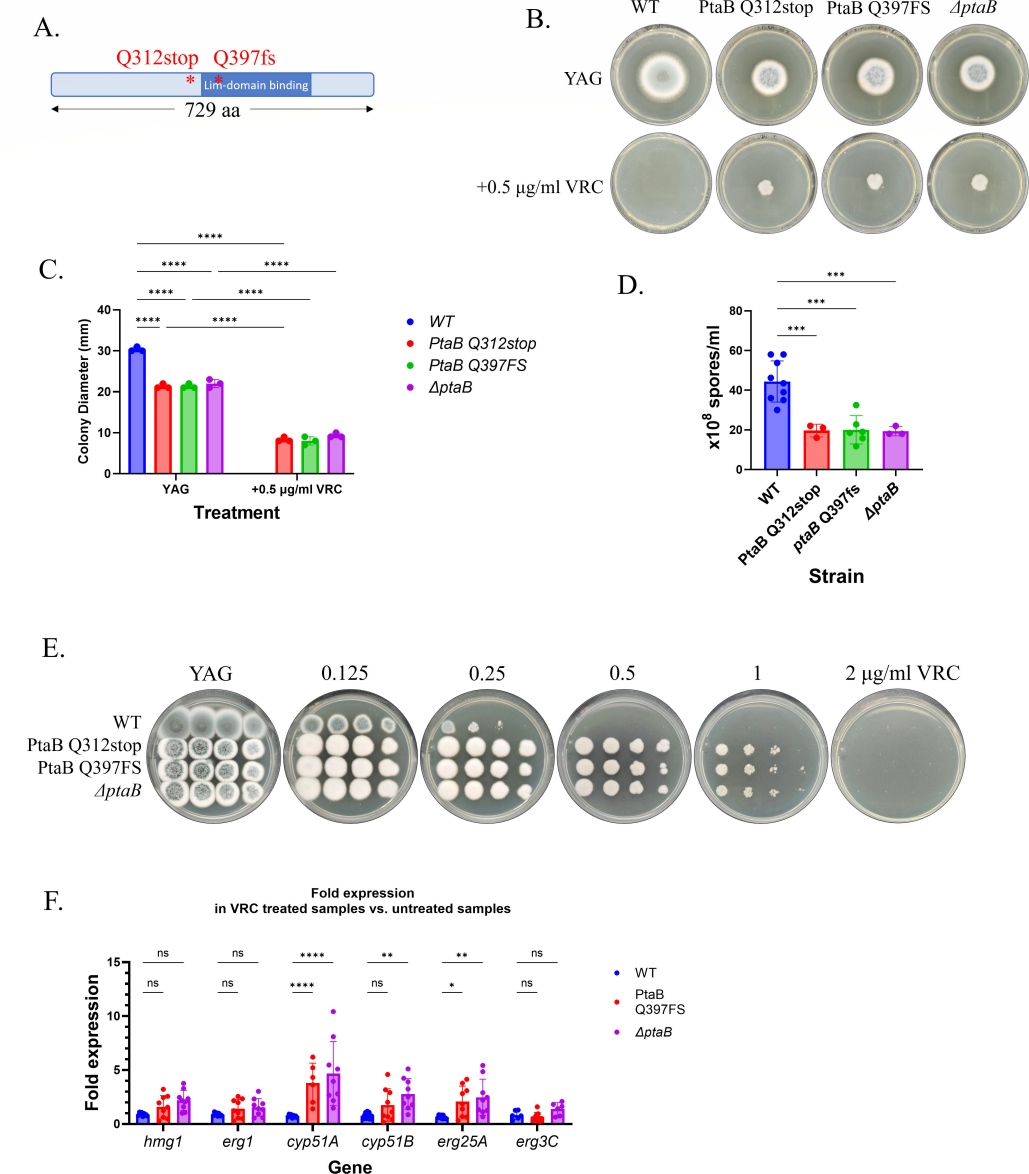
*PtaB* mutation or deletion contributes to triazole resistance and increases the expression of key ergosterol biosynthesis genes. (**A**) Schematic representation of PtaB showing the positions of Q312stop and Q397FS mutations located before and within the LIM-binding domain, respectively. (**B**) Point inoculation of PtaB Q312stop, PtaB Q397FS, and *ΔptaB* on YAG agar with or without VRC, incubated for 72 h at 37°C. (**C**) Radial growth measurements of strains *ptaB* mutants show significantly reduced colony diameter compared to the control WT strain (****, *P* < 0.0001). Error bars indicate the standard deviation of biological triplicate. (**D**) Conidiation assays revealing significantly reduced sporulation in the *ptaB* mutants (*P <* 0.001). Error bars represent the standard deviation of biological replicates. (**E**) Serial dilution assays of WT, strains PtaB Q312stop, PtaB Q397FS, and *ΔptaB* on YAG agar containing increasing concentrations of VRC. (**F**) qPCR analysis of *hmg1*, *erg1*, *cyp51A*, *cyp51B*, *erg25A,* and *erg3C* expression in the WT, *ΔptaB,* and PtaB Q397FS strains, grown with and without VRC. Error bars indicate the standard deviation of three technical replicates of three biological experiments. * *P* < 0.05, ** *P* < 0.01, *** *P* < 0.001, **** *P* < 0.0001, ns, not significant.

*PtaB* encodes a transcriptional activator of biofilm formation and conidiation in *A. fumigatus* (47, 48). We identified a PtaB frameshift mutation (Q397FS) in the LIM-binding domain in strains C12 and D12 (WT background) and a PtaB Q312stop mutation preceding the Lim-binding domain in strain B12 (*Δcyp51A* background) (Fig. 6A). We introduced PtaB Q397FS and PtaB Q312stop to replace WT*-ptaB* in the WT parental strain, generating strains PtaB Q397FS and PtaB Q312stop, respectively. We also generated a strain in which *ptaB* was deleted (*ΔptaB*). PtaB Q397FS, PtaB Q312stop, and *ΔptaB* showed decreased conidiation and radial growth, as previously described for the *ΔptaB* strain (Fig. 6B through D) (48). The mutant strains showed a fourfold increase in VRC MIC on YAG agar plates (Fig. 6E) and a fourfold increase in VRC/ITC/POS/ISV MICs by broth microdilution (Table 3). Interestingly, PtaB Q397FS, PtaB Q312stop, and *ΔptaB* showed increased sensitivity (fourfold reduction in MEC) to CAS, suggesting decreased cell wall integrity.

We hypothesized that PtaB is a negative transcriptional regulator of ergosterol pathway gene expression, leading to upregulation and decreased triazole susceptibility when *ptaB* is deleted. To test this, we performed qPCR analysis of *hmg1*, *erg1*, *cyp51A*, *cyp51B*, *erg25A,* and *erg3C* expression in the WT, *ΔptaB,* and PtaB Q397FS strains grown for 20 h in YAG with and without VRC (0.5 MIC) for 4 h at 37°C. The results show a significant increase in *cyp51A* (*P* < 0.0001), *cyp51B* (*P* < 0.05 for *ΔptaB*), and *erg25A* (*P* < 0.05) expression in the *ΔptaB* and PtaB Q397FS strains in the presence of VRC (Fig. 6F), suggesting that PtaB is a negative transcriptional regulator of key ergosterol biosynthesis genes.

### Correction of the SrbA L250P substitution in the parental resistant strain leads to decreased resistance

**Fig 7:**
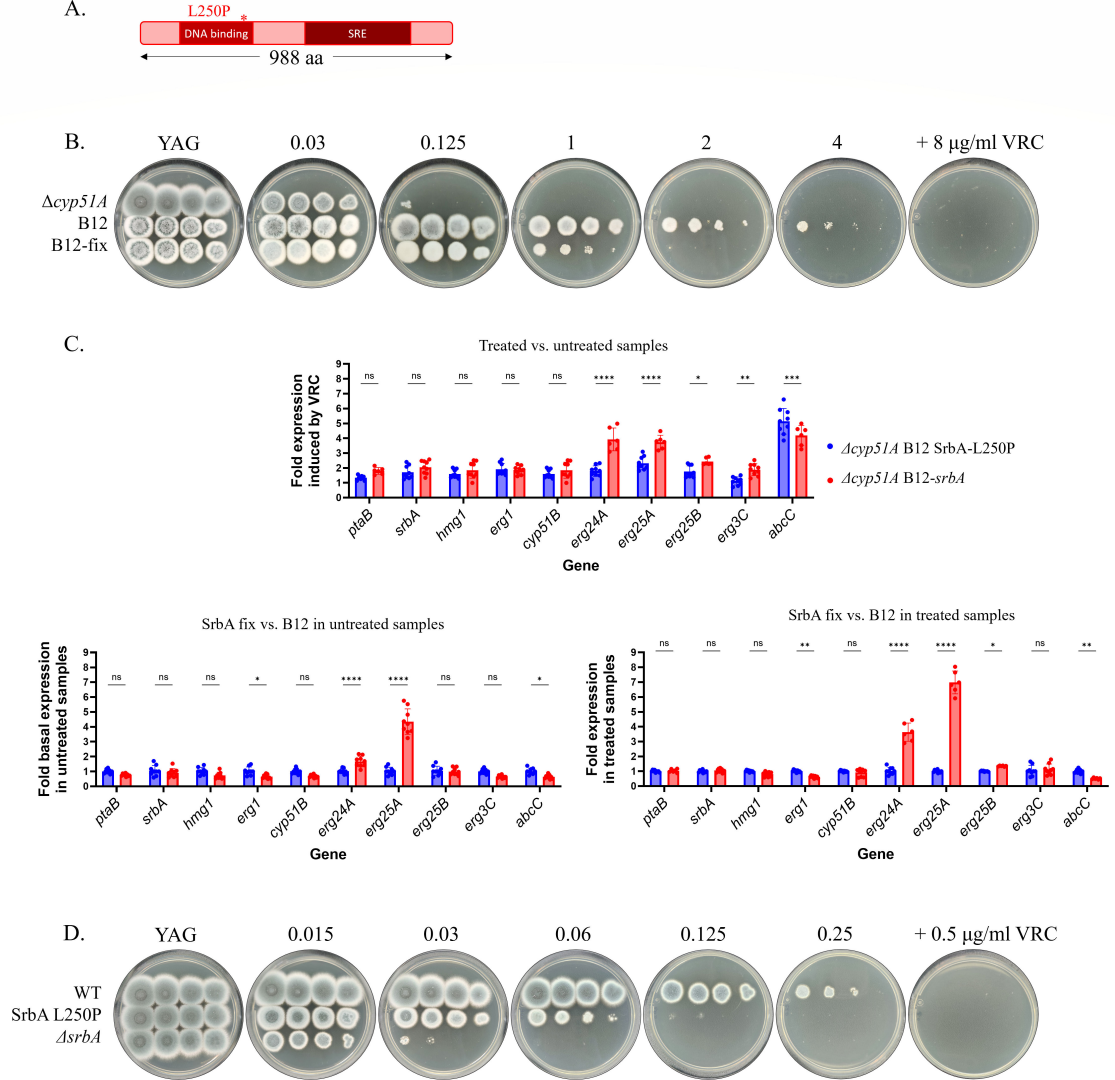
The SrbA L250P substitution modulates voriconazole resistance. (**A**) Schematic representation of SrbA showing the position of the L250P substitution within the DNA-binding domain. (**B**) Serial dilution assays of parental *Δcyp51A*, the evolved parental *Δcyp51A* B12 SrbA-L250P strain, and *Δcyp51A* B12-*srbA* in which the SrbA L250P mutation was corrected. Strains were grown on YAG agar containing increasing concentrations of VRC. (**C**) qPCR analysis of *ptaB*, *srbA*, *hmg1*, *erg1*, *cyp51B*, *erg24A, erg25A, erg25B, erg3C,* and *abcC* expression in *Δcyp51A* B12 SrbA-L250P and *Δcyp51A* B12-*srbA* strains, grown with and without VRC treatment. Error bars indicate the standard deviation of three technical replicates of three biological experiments. (**D**) Serial dilution assays of WT, *srbA* L250P, and *ΔsrbA* on YAG agar with increasing VRC concentrations. * *P* < 0.05, ** *P* < 0.01, *** *P* < 0.001, **** *P* < 0.0001, ns, not significant.

The sterol-regulated transcription factor SrbA plays a key role in the regulation of sterol biosynthetic genes, and its deletion leads to azole sensitivity (14, 49, 50). We identified an SrbA mutation (L250P) in the DNA-binding domain in the evolved strain *Δcyp51A* B12 (Fig. 7A). We introduced a WT *srbA* allele into *Δcyp51A* B12 SrbA-L250P, replacing the mutated allele and generating strain *Δcyp51A* B12-*srbA*. This *srbA*-corrected strain showed a twofold decrease in susceptibility to VRC on YAG agar plates compared to the parental evolved *Δcyp51A* B12 *srbA*-L250P strain (Fig. 7B) and a twofold decrease in VRC MIC by broth microdilution (Table 4). This result indicates that the SrbA L250P substitution is a positive driver of triazole resistance, as its correction in the parental evolved B12 strain leads to increased triazole susceptibility. We hypothesized that the SrbA L250P substitution leads to altered expression of genes in the ergosterol biosynthesis pathway. To test this, we compared the expression of key genes in the ergosterol biosynthetic pathway between *Δcyp51A* B12 *srbA*-L250P and the corrected strain *Δcyp51A* B12-*srbA*, grown for 20 h in YAG with and without VRC (0.5 MIC) for 4 h at 37°C (Fig. 7C). Results showed that the expression of *erg24A* and *erg25A* was significantly increased in the corrected strain *Δcyp51A* B12-*srbA*-WT compared to the evolved *Δcyp51A* B12 strain. Overexpression of *erg24A* and *erg25A* under VRC treatment could cause an accumulation of toxic eburicol-derived sterols (see Discussion).

We also introduced the SrbA (L250P) substitution into the WT parental strain to generate SrbA L250P. Surprisingly, we found that strain SrbA L250P showed a twofold decrease in VRC MIC on YAG agar plates (Fig. 7D) and a twofold decrease in VRC MICs by broth microdilution (Table 3). This indicates that the SrbA L250P substitution generates triazole sensitivity in the WT background and contributes to triazole resistance in the genetic background of strain *Δcyp51A* B12 SrbA-L250P, possibly due to epistatic interactions with other mutated genes in the B12 background.

### Mutations in *hmg1* and *ptaB* emerged during the early stages of the evolutionary process

**Fig 8:**
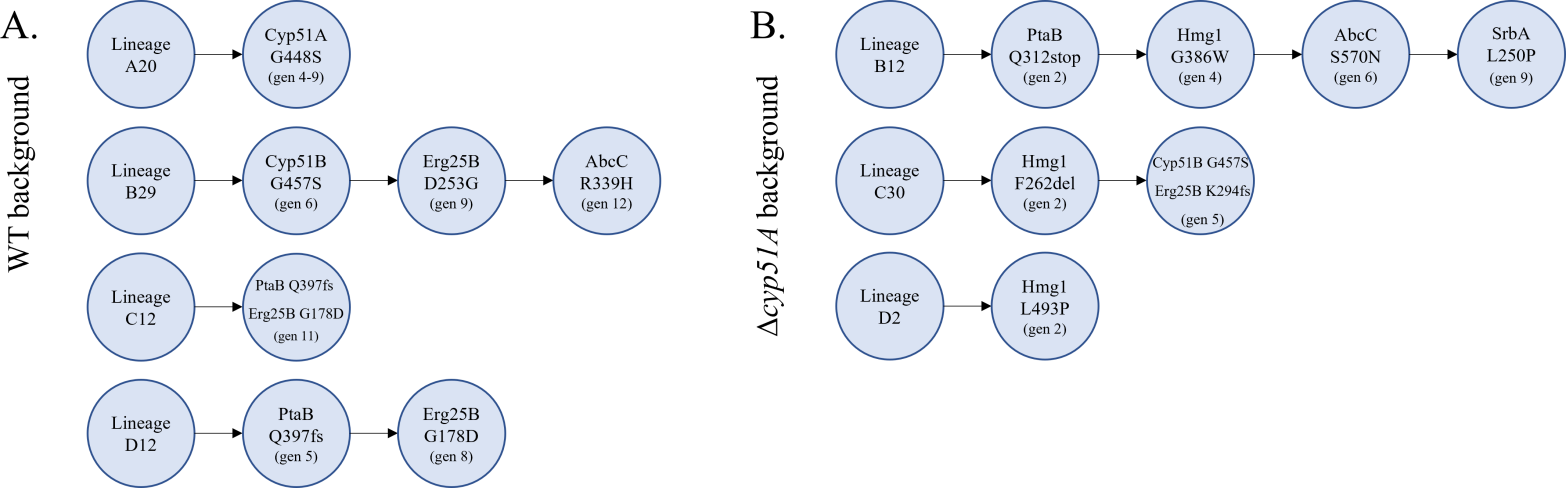
Mutation timeline in lineages evolved from the (**A**) WT and (**B**) Δcyp51A backgrounds. ARMS-PCR and Sanger sequencing showed early appearance of hmg1 (generations 2–4) and ptaB (generations 2–5) mutations, intermediate cyp51B (generation 6), and later abcC (generations 6, 12), erg25B (generations 8, 9), and srbA (generation 9) mutations.

To identify the timeline of the occurrence of mutations in the evolved strains, we analyzed isolates collected at each passage of the experimental evolution using ARMS-PCR followed by Sanger sequencing (Fig. 8). *Hmg1* mutations appeared early, specifically in generation 4 of lineage B12 and generation 2 of lineages C30 and D2. Similarly, *ptaB* mutations were detected early, in generation 5 of lineage D12 and generation 2 of lineage B12. Mutations in *cyp51B* (generation 6) arose during an intermediate stage, whereas mutations in *abcC* (generations 6 and 12), *erg25B* (generations 8 and 9), and *srbA* (generation 9) appeared later in the evolutionary timeline. These findings, however, do not fully account for the acquisition of resistance observed in the WT background strains during the earliest passages (passages 1–6), nor do they explain the progressive resistance observed in strains B29 and C30 during later stages of evolution (see Discussion).

### Missense mutations in *hmg1, ptaB*, *abcC,* and *srbA* are more prevalent in clinical azole-resistant strains

Finally, to determine whether mutations in the genes identified in this study are present in *A. fumigatus* clinical isolates, we performed a bioinformatics analysis of 351 publicly available genomes, including 170 azole-susceptible strains, 125 *cyp51A*-based resistant strains, and 56 resistant strains lacking *cyp51A* mutations. Missense mutations in SrbA (F105L) and AbcC (N1213D) were more frequent in Cyp51A-independent resistant strains than in susceptible strains, according to odds ratio analysis (Table 5), suggesting a potential contribution to azole resistance. In addition, mutations in Hmg1 (E105K), PtaB (G34A), AbcC (R166G), and SrbA (L28del) were significantly enriched in Cyp51A-dependent resistant strains, suggesting a potential role for these genes in facilitating azole adaptation.

**TABLE 5:** Prevalence of genetic mutations and association with phenotypic groups^*a*^^,^^*b*^

Gene	Scaffold (chromosome)	Position	Nucleotide change and ORF position	Amino acid change	Mutation type	Isolates with variant			Odds ratio		*P*-value	
						S	R−	R+	S vs R−	S vs R+	S vs R−	S vs R+
AFUA_1G14330 (*abcC/cdr1B*)	NC_007194.1	3836133	3637A>G	N1213D	mis.	1	2	0	**0.16**	NA	0.153	1.000
AFUA_2G01260 (*srbA*)	NC_007195.1	297209	313T>C	F105L	mis.	45	24	6	**0.48**	7.1	0.0291*	<0.001*
AFUA_2G03700 (*hmg1*)	NC_007195.1	985246	313G>A	Glu105Lys	mis.	5	2	28	0.82	**0.11**	1.000	<0.001*
AFUA_2G12910 (*ptaB*)	NC_007195.1	3311532	197A>G	Q66R	mis.	0	2	0	0	NA	0.060	NA
AFUA_2G12910 (*ptaB*)	NC_007195.1	3311628	101G>C	G34A	mis.	43	4	64	4.38	**0.32**	0.0039*	<0.001*
AFUA_2G12910 (*ptaB*)	NC_007195.1	3310621	1029G>A	M343I	mis.	44	4	65	4.52	**0.32**	0.0023*	<0.001*
AFUA_1G14330 (*abcC/cdr1B*)	NC_007194.1	3832726	496C>G	R166G	mis.	2	0	8	NA	**0.18**	1.000	0.0203*
AFUA_2G01260 (*srbA*)	NC_007195.1	296976	81_83delCTT	L28del	del.	3	0	6	NA	**0.36**	1.000	0.175

^*a*^ S, susceptible (*n* = 170); R−, resistant non-Cyp51A (*n *= 56); and R+, resistant Cyp51A based (*n *= 125); mis., missense mutation; del., deletion; NA, not available (could not be calculated). Odds ratio > 1 indicates association with the first group listed in the comparison. Odds ratios < 0.5 are highlighted in bold.

^*b*^ *P-values < 0.05. Odds ratios and *P*-values were calculated using Fisher's exact test to compare the distribution of each mutation between groups.

## DISCUSSION

In this study, we used experimental evolution of *A. fumigatus* under increasing VRC concentrations to find novel mechanisms of triazole resistance. Whole-genome sequencing and functional validation identified novel resistance-conferring mutations in *hmg1*, *abcC, erg25B*, *ptaB*, and *srbA*, in addition to the well-characterized mutations in *cyp51A*, *cyp51B*, and *hmg1* (6, 15, 18, 19, 21–25). Mutations in *ptaB*, *erg25B*, *abcC,* and *srbA* add new contributors to resistance, beyond the usual drug target changes and efflux regulation.

### *Hmg1* mutations extend beyond the sterol-sensing domain

Consistent with earlier reports, we found that *hmg1* is a frequent early target of selection under azole pressure (21–25). Previous studies identified resistance mutations clustering within the sterol-sensing domain, altering the feedback regulation of sterol synthesis (51). Surprisingly, our work shows that the L493P substitution, located outside the SSD, also contributes to resistance. This indicates that Hmg1-dependent resistance mechanisms extend beyond the known SSD mutations and may involve broader impacts on enzyme stability or regulatory interactions. Notably, *hmg1* mutations were exclusively selected in the Δ*cyp51A* background, suggesting compensatory rewiring of ergosterol biosynthesis when the primary azole target is absent.

### Efflux transporter *abcC* mutation contributes modestly to resistance

The novel R339H mutation identified in the ATPase domain of AbcC conferred mild VRC resistance. While AbcC overexpression has previously been associated with clinical azole resistance (20), our findings demonstrate for the first time that in *A. fumigatus*, point mutations in *abcC* can also contribute to resistance. The underlying mechanism remains unresolved: the mutation may enhance protein stability and thus increase expression levels, or alternatively, directly augment efflux activity without changes in expression. Similar categories of mutations—affecting either protein abundance or transporter efficiency—have been reported in experimentally evolved azole-resistant yeast strains (52, 53).

### Erg25B mutations reduce the accumulation of methylated sterol intermediates

Our findings provide evidence that erg25B loss-of-function or missense mutations can contribute to azole resistance. Sterol profiling showed that *Δerg25B* and Erg25B D253G mutants accumulate markedly reduced levels of the 14-methyl sterol diol under VRC treatment. The buildup of methylated sterols has previously been implicated in toxicity in both *Candida* spp. and *A. fumigatus* (47, 54). However, recent work by Elsaman et al. (54) demonstrated that in *A. fumigatus*, eburicol—and not the downstream methylated sterols—is the key toxic intermediate responsible for azole fungicidal activity. Since eburicol accumulation is not reduced in our *erg25B* mutants, the decreased azole susceptibility cannot be explained by lower diol levels and must occur through a mechanism independent of diol-associated toxicity. The precise mechanism by which Erg25B deletion contributes to resistance remains to be determined.

### PtaB links morphogenesis, transcriptional regulation, and resistance

We show that frameshift and nonsense mutations in *ptaB*, encoding a LIM-binding-domain protein involved in biofilm formation and conidiation (47, 48), contribute to triazole resistance. Mutant and Δ*ptaB* strains displayed increased expression of *cyp51A*, *cyp51B*, and *erg25A*, implicating PtaB as a negative regulator of sterol biosynthetic genes. The dual phenotype of impaired conidiation with enhanced resistance highlights the trade-offs that may shape *ptaB*-mutant survival in clinical vs environmental niches. Importantly, *ptaB* mutations were also enriched in resistant clinical isolates, suggesting translational relevance.

### SrbA mutations exhibit context-dependent effects

SrbA, the sterol regulatory element-binding protein, which is an important contributor to azole resistance (14, 49, 50), emerged as a late target of selection. The L250P substitution enhanced resistance in the evolved Δ*cyp51A* B12 background but conferred sensitivity in a WT background, indicating potential epistatic interactions with other mutations. These findings highlight the complexity of SrbA’s regulatory role, which may change between resistance and sensitivity depending on the genetic context. In the ergosterol pathway, SrbA directly binds and activates several key genes, including *cyp51A, cyp51B, erg25A*, and *erg24A* (14, 49, 50). When we corrected the L250P mutation in the evolved *Δcyp51A* B12 strain, we observed significantly increased expression of *erg24A* and *erg25A*, suggesting that SrbA L250P shows reduced DNA binding and transcriptional activation of these genes compared to WT SrbA. Consistent with this, the SrbA-corrected strain showed reduced azole resistance. Under azole treatment, inhibition of Cyp51A and Cyp51B leads to accumulation of eburicol, which can then be metabolized into toxic eburicol-derived sterols by Erg24A and Erg25A. Thus, elevated *erg24A*/*erg25A* expression in the corrected strain may promote the buildup of these toxic intermediates, providing a mechanistic explanation for the greater azole sensitivity observed upon SrbA correction. However, this explanation requires additional experimentation, such as inducible overexpression of *erg24A* and *erg25A*, to determine whether this is indeed the underlying mechanism.

### Evolutionary dynamics and clinical parallels

Tracking mutations over the course of experimental evolution revealed that *hmg1* and *ptaB* mutations arise early, followed by *cyp51B* and *abcC* mutations, while *erg25B* and *srbA-*specific mutations occur later. This suggests a sequential adaptation strategy, with initial resistance mediated by metabolic rewiring and later reinforced by transcriptional or efflux-related changes. Bioinformatic analysis of clinical genomes revealed a statistically significant enrichment of *ptaB*, *hmg1*, *abcC*, and s*rbA* missense mutations in azole-resistant isolates; however, many susceptible isolates also carried mutations in these genes, and the specific variants identified experimentally here were not observed among clinical isolates. Thus, while these parallels between experimental evolution and clinical genomic patterns are suggestive and support the potential biological relevance of these pathways, they do not establish a direct causal link between individual mutations and clinical resistance, and further functional validation will be required.

### Unexplained resistance in early and late passages

While our mutation timeline provides a framework for the stepwise acquisition of resistance, it does not fully explain the resistance observed in WT strains during the earliest passages (generations 1–6), nor the gradual increase in resistance seen in strains B29 and C30 at later stages. One possibility is that early resistance arises from transient, non-genetic adaptations such as changes in gene expression, chromatin remodeling, or altered stress responses that allow survival under low drug concentrations before stable mutations are acquired. Similar transient tolerance mechanisms have been reported in fungi exposed to antifungals and other stresses (6, 30, 31). In the case of B29 and C30, progressive increases in resistance may reflect polygenic adaptation, with multiple low-impact mutations and potential copy number variations or regulatory adjustments acting additively. It is also possible that mutations outside of our candidate genes, including those in non-coding regions or affecting global regulators, contributed to resistance but were not prioritized in our focused analysis. These observations highlight that experimental evolution can capture both genetic and non-genetic layers of adaptation and suggest that azole resistance in *A. fumigatus* may involve a continuum from reversible tolerance to stable, mutation-driven resistance.

### Conclusion

Collectively, our work identifies novel genes and pathways contributing to triazole resistance in *A. fumigatus* and demonstrates the utility of experimental evolution for uncovering clinically relevant mechanisms (30, 31). By showing that changes in resistance can emerge via mutations in transcriptional regulators (*ptaB* and *srbA*), sterol biosynthesis (*erg25B* and *hmg1*), and efflux transporters (*abcC*), we broaden the mechanistic understanding of azole resistance. These insights may have implications for surveillance, resistance diagnostics, and the rational design of antifungal strategies. Future studies should examine the prevalence of these mutations across global clinical collections and their impact on *in vivo* virulence and treatment outcomes.

## Data Availability

The WGS data were submitted to NCBI under BioProject ID PRJNA1347423.

## SUPPLEMENTAL MATERIAL

Supplemental material aac.01635-25-s0001.docx Tables S1 to S6; Fig. S1 to S5.

ASM does not own the copyrights to Supplemental Material that may be linked to, or accessed through, an article. The authors have granted ASM a non-exclusive, world-wide license to publish the Supplemental Material files. Please contact the corresponding author directly for reuse.
