# Supplementary material for: Directed evolution of the pathogenic mold Aspergillus fumigatus reveals novel genes contributing to triazole resistance
Source: Antimicrob Agents Chemother. 2026 Mar 4;70(4):e01635-25. doi: 10.1128/aac.01635-25 (PMC13041316; doi:10.1128/aac.01635-25)
Supplement: Supplemental material — Tables S1 to S6; Fig. S1 to S5. [file aac.01635-25-s0001.docx]

**Supplemental Material and Tables.**

**Construction and verification of *hmg1* L262del/ G386W/ L493P mutant strains.** Primers "Hmg1-F" and "Hmg1-R" (Table S3) were used for amplification of the mutated *hmg1* gene, with 50 bp flanking the 3' and 5' ends of the gene (Figure S1.A). The amplicon was introduced into the *ΔKU80* strain, along with two gRNAs, one for each 5' and 3' ends of the target gene (Table S4), and Cas9 enzyme (IDT) and pTel-hyg^R^. The transformation plates contained 0.5 μg/ml VRC, after which VRC-resistant selected colonies were streaked twice on YAG agar plates for strain purification. F262del and L493P mutants were sent directly to sequencing. G386W mutants were verified by ARMS-PCR with primers set to amplify the gene (primers "Hmg1-386-OF" and "Hmg1-386-OR"), the WT sequence when present ("Hmg1-386-WT-R" and "Hmg1-386-OF") or the mutated sequence when present ("Hmg1-386-M-F" and "Hmg1-386-OR") (Table S5 and Figure S1.B). All final isolates displaying the correct band pattern were then verified by sequencing (Figure S1.C-E).


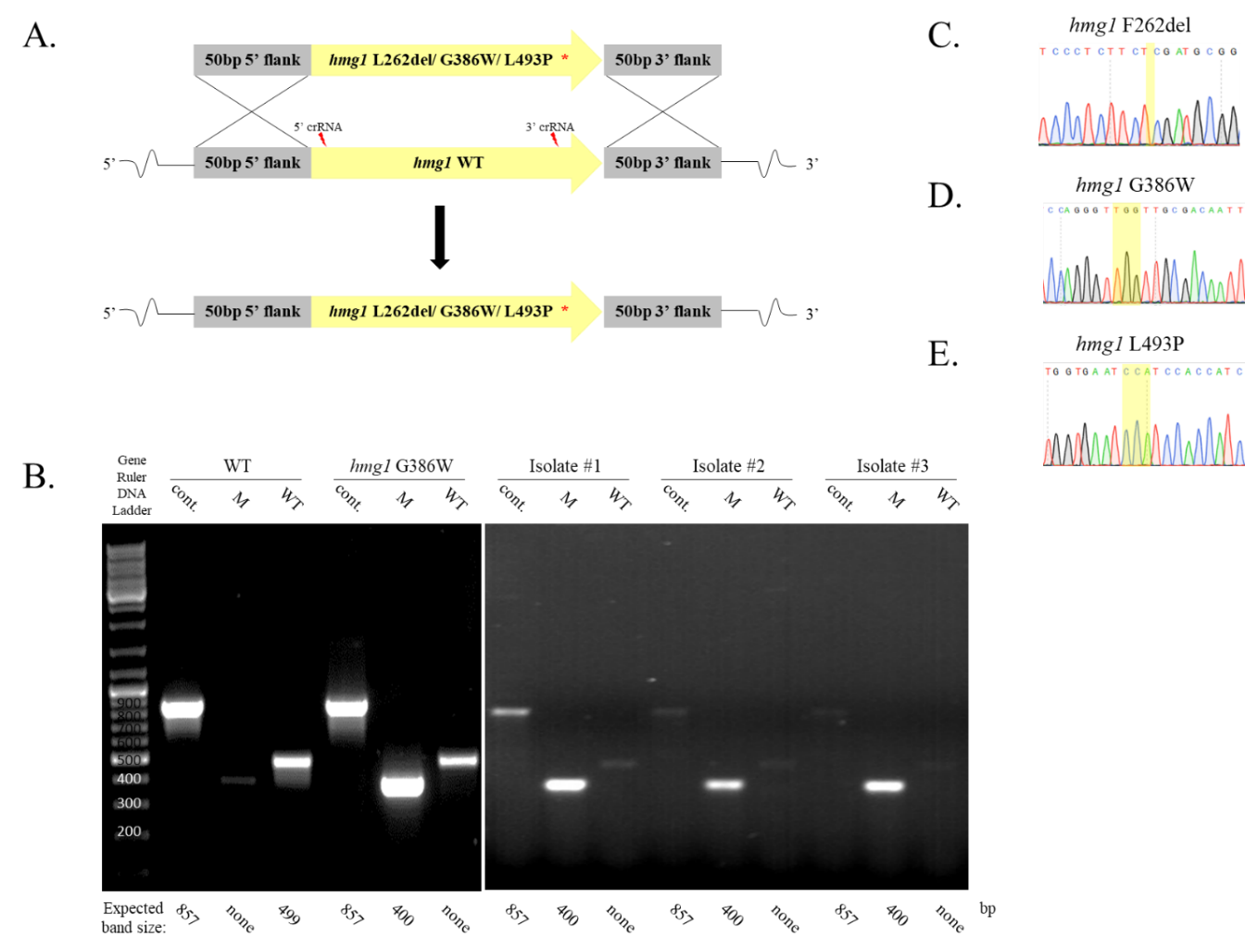


**Figure S1.** Generation and verification of *hmg1* L262del/ G386W/ L493P mutant strain.

A. Schematic of gene replacement design. To introduce the *hmg1* mutation, the PCR-amplified mutated *hmg1* gene was used as a repair template, transformation was performed with pTel-hyg^R^ and two guide crRNAs *hmg1* 5’ and *hmg1* 3'. Similar methodology was used to generate all *hmg1* gene replacements described. B. ARMS-PCR analysis for the G386W mutation of one negative control isolate ("WT"), showing the control and "WT" bands, and a very faint "M" (Mutant) band, a *hmg1* G386W mutant (the original evolved strain in which this mutation was originally found) showing the control and both the "M" and "WT" bands, and three isolates transformed with *hmg1* G386W repair template, all showing the "M" band. Strains were subsequently verified by Sanger sequencing, C. A "TCT" deletion from the *hmg1* sequence in F262del, D. a *hmg1* sequence of "Tgg" instead of "Ggg" for G386W, and E. "Tgg" instead of "Ggg" for G386W.

**Construction and verification of *abcC* R339H mutant strain.** Primers "AbcC-F" and " AbcC -R" (Table S3) were used for amplification of the mutated *abcC* gene, with 50 bp flanking the 3' and 5' ends of the gene (Figure S2.A). The amplicon was introduced into the *ΔKU80* strain, along with two gRNAs, one for each 5' and 3' ends of the target gene (Table S2), and Cas9 enzyme (IDT) and pTel-hyg^R^. Transformant colonies were screened on YAG + 0.25 μg/ml VRC agar plates, after which selected colonies were streaked twice on YAG agar plates for strain purification. Mutants were verified by ARMS-PCR with primers set to amplify the gene (primers "AbcC-F" and " AbcC OR"), the WT sequence when present ("AbcC WT-R" and " AbcC-F") or the mutated sequence when present ("AbcC M-F" and "AbcC OR") (Table S5 and Figure S2.B). The final isolates displaying the correct band pattern were then verified by sequencing (Figure S2.C).


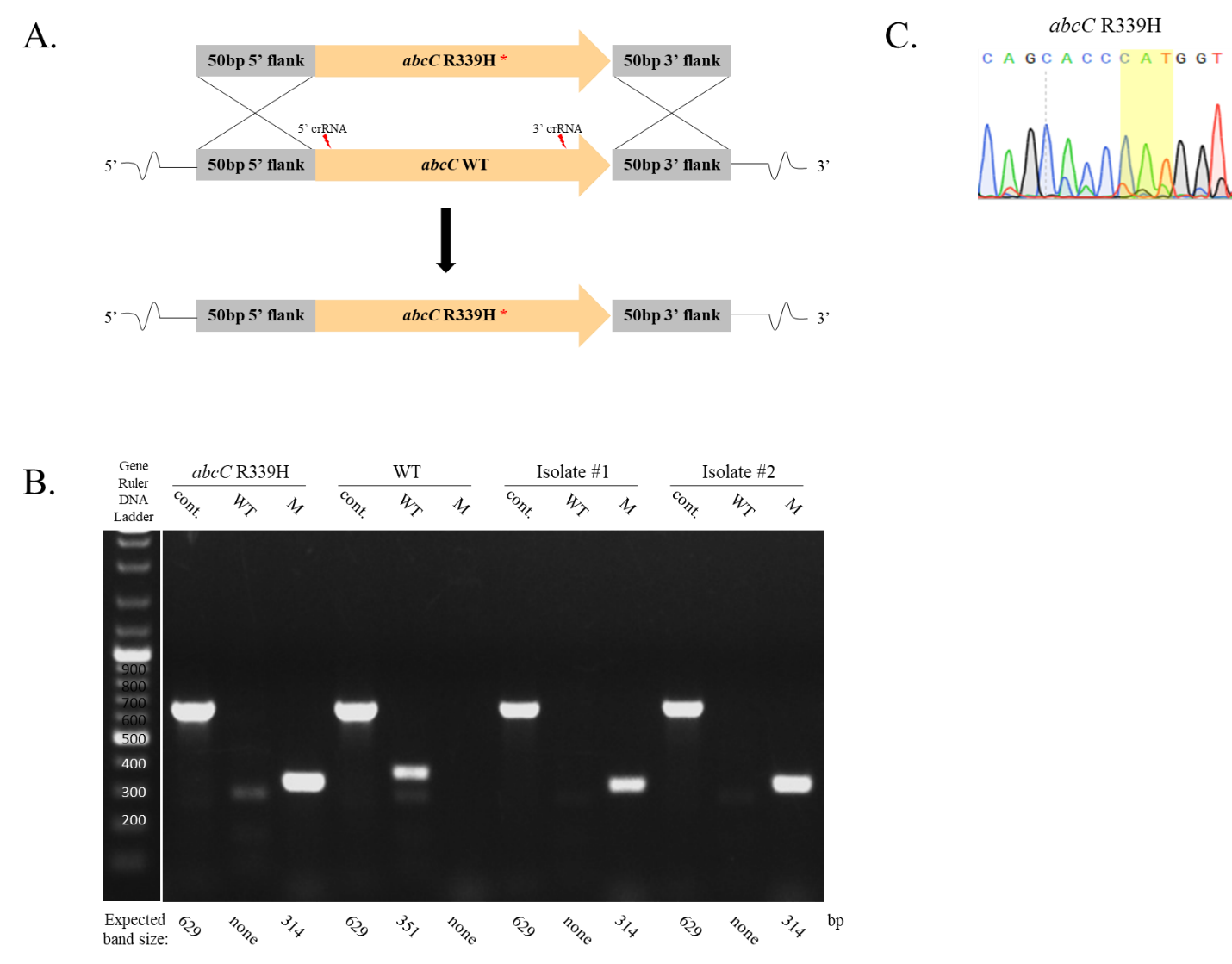


**Figure S2.** Generation and verification of *abcC* R339H mutant strain.

A. Schematic of gene replacement design. To introduce the *abcC* mutation, the PCR-amplified mutated *abcC* gene was used as a repair template, transformation was performed with pTel-hyg^R^ and two guide crRNAs *abcC* 5’ and *abcC* 3'. Similar methodology was used to generate all *abcC* gene replacements described. B. ARMS-PCR analysis of one negative control isolate ("WT"), showing the control and "WT" bands, but not the "M" (Mutant) band, a *abcC* R339H mutant (the original evolved strain in which this mutation was originally found) showing the control and the "M" bands, and two isolates transformed with *abcC* R339H repair template, showing the control and the "M" bands. C. Strains were subsequently verified by Sanger sequencing, an *abcC* sequence of "cAt" instead of "cGt".

**Construction and verification of *erg25B* D253G and *erg25B* WT strains, and *erg25B*-null strain.** Primers "Erg25B-F" and "Erg25B-R" (Table S3) were used for amplification of the mutated and WT *erg25B* gene, with 50 bp flanking 5' end of the gene, and primers "PtrA (Erg25B)-F" and "PtrA (Erg25B)-R" were used for amplification of the PtrA cassette, with 50 bp flanking 3' end of the *erg25B* gene. The two amplicons were then fused by overlap-PCR and the final construct was used as a repair template (Figure S3.A). Primers "Erg25B KO-F" and "Erg25B KO-R" (Table S3) were used for amplification of the PtrA cassette, with 50 bp flanking the 3' and 5' ends of the *erg25B* gene (Figure S3.B). The amplicons were introduced into the *ΔKU80* strain, along with two gRNAs, one for each 5' and 3' ends of the target gene (Table S4), and Cas9 enzyme (IDT). Transformant colonies were screened on AMM + 0.1 μg/ml PtrA agar plates, after which selected colonies were streaked twice on YAG agar plates for strain purification. *erg25B* D253G and *erg25B* WT isolates were verified by ARMS-PCR with primers set to amplify the gene (primers "Erg25B OF" and "Erg25B-R"), the WT sequence when present ("Erg25B WT-R" and "Erg25B OF") or the mutated sequence when present ("Erg25B M-F" and "Erg25B-R") (Table S3 and Figure S3.C). erg25B-null isolates were verified by PCR (data not shown). The final isolates were then verified by sequencing (Figure S3.D-E).


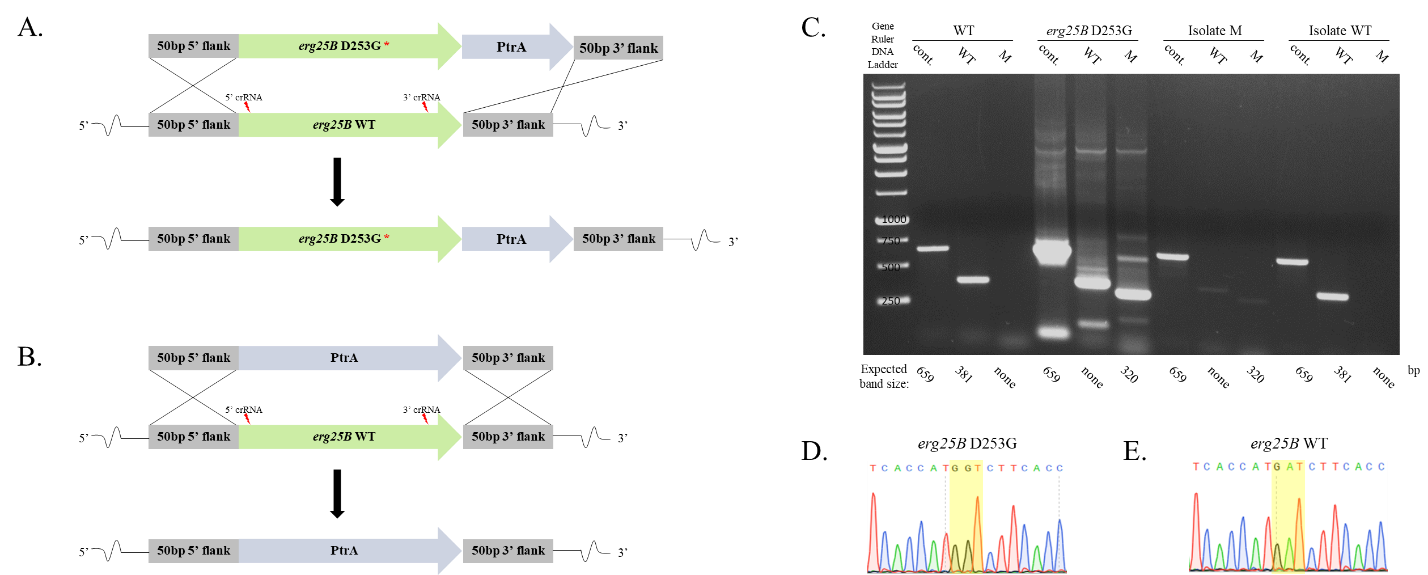


F.
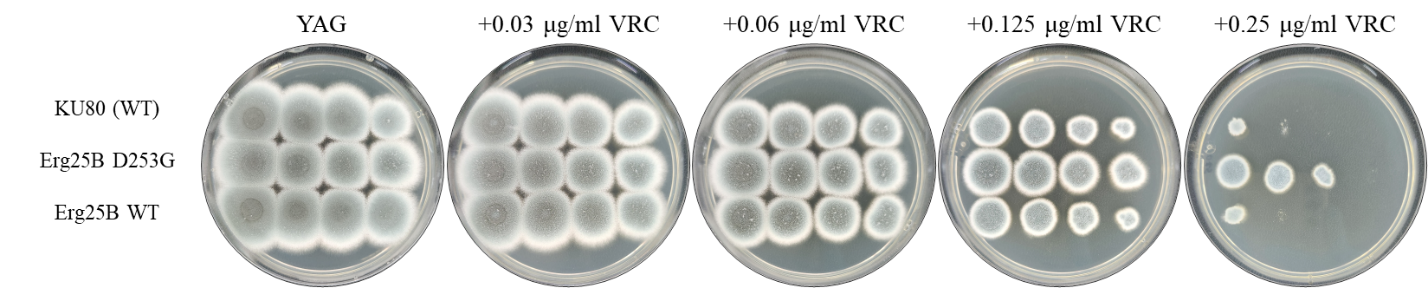


**Figure S3.** Generation and verification of *erg25B* D253G and *erg25B* WT strains, and *erg25B*-null strain.

A. Schematic of gene replacement design. To introduce the *erg25B* mutation or WT gene with PtrA, the *erg25B* mutated gene and the PtrA cassette were PCR amplified, fused by overlap-PCR, and the final construct was used as a repair template. Transformation was performed with two guide crRNAs *erg25B* 5’ and *erg25B* 3'. Similar methodology was used to generate all *erg25B* gene replacements described. B. To create the erg25B-null strain, the PCR amplified PtrA cassette was used as a repair template. Transformation was performed with two guide crRNAs *erg25B* 5’ and *erg25B* 3'. C. ARMS-PCR analysis of one negative control isolate ("WT"), showing the control and "WT" bands, but not the "M" (Mutant) band, the *erg25B* D253G repair template showing the control and both the "M" and "WT" bands, and two isolates transformed with *erg25B* D253G repair template, one that integrated the entire repair template with the mutated *erg25B* gene, showing the control and the "M" and "WT" bands, and one that integrated only the PtrA segment of the repair template, showing the control and the "WT" bands. Strains were subsequently verified by Sanger sequencing, showing D. An *erg25B* sequence of "gGt" instead of "gAt" for the D253G mutant, and E. A WT sequence of "gAt" for the WT isolate. (F) Serial dilution assays of parental KU80 (WT), strain Erg25B D253G in which the Erg25B D253G mutation was inserted into the *erg25B* locus to mutate the WT gene, and Erg25B WT (control transformed with WT-*erg25B*). Strains were grown on YAG agar containing increasing concentrations of VRC.

**Construction and verification of *srbA* L250P strains.** Primers "SrbA-F" and "SrbA (PtrA)-R" or "SrbA (hph)-R" (Table S3) were used for amplification of the mutated or WT *srbA* gene, with 50 bp flanking 5' end of the gene, and primers "PtrA (SrbA)-F" and "PtrA (SrbA)-R" or "hph (SrbA)-F" and "hph (SrbA)-R" were used for amplification of the PtrA or hyg cassettes, with 50 bp flanking 3' end of the *srbA* gene. The two amplicons were then fused by overlap-PCR and the final constructs were used as a repair template (Figure S4.A). Similar methodology was used to correct the mutated *srbA* L250P gene in the *Δcyp51A* B12 strain to a WT copy (Figure S4.B). The amplicons were introduced into the *ΔKU80* strain, along with two gRNAs, one for each 5' and 3' ends of the target gene (Table S4), and Cas9 enzyme (IDT). Transformant colonies were screened on AMM + 0.1 μg/ml PtrA or YAG + 350 μg/ml hyg agar plates, after which selected colonies were streaked twice on YAG agar plates for strain purification. *srbA* L250P isolates were verified by ARMS-PCR with primers set to amplify the gene (primers "SrbA OF" and "SrbA -R"), the WT sequence when present ("SrbA WT-F" and "SrbA OR") or the mutated sequence when present ("SrbA M-R" and "SrbA-OF") (Table S5 and Figure S4.C). The final isolates were then verified by sequencing (Figure S4.D-E).


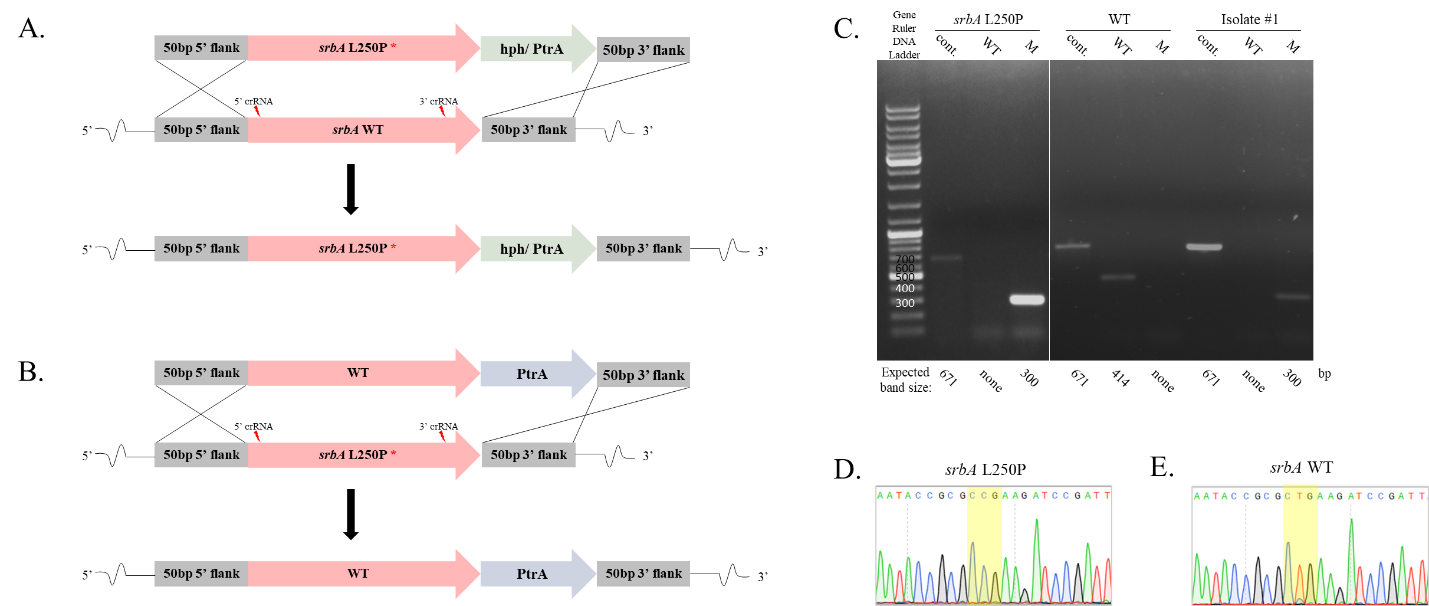


E. **
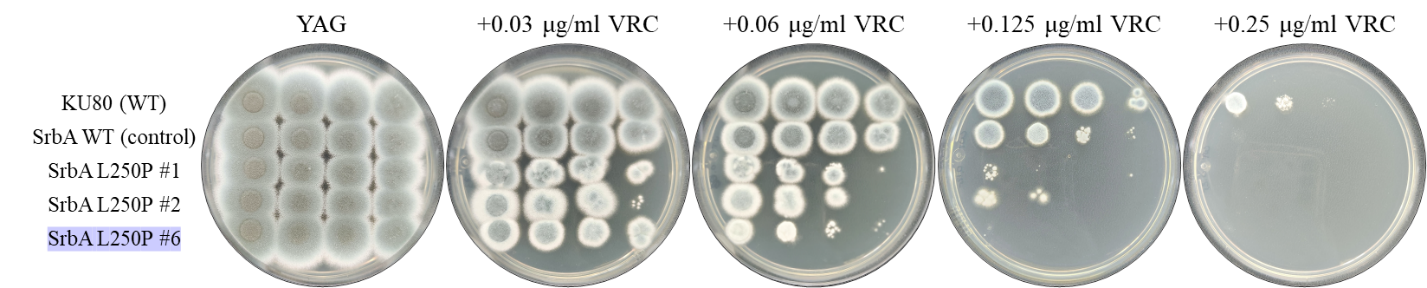
**

F.
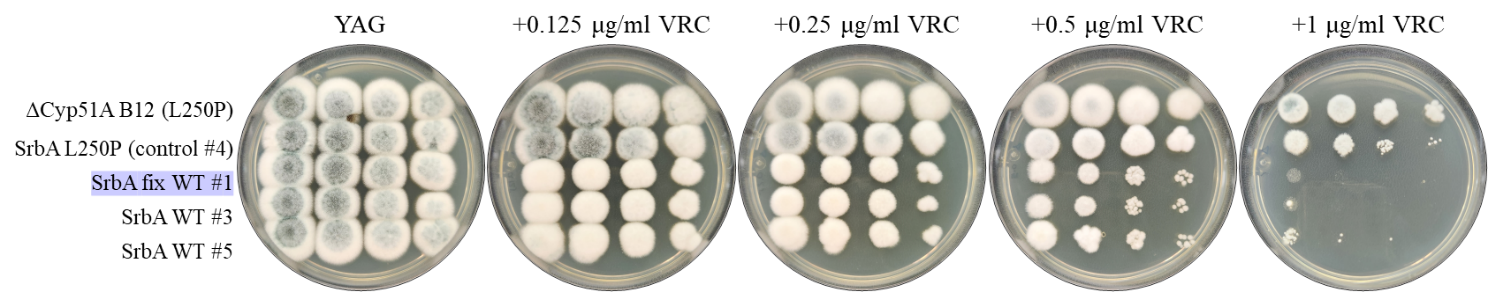


**Figure S4.** Generation and verification of *srbA* L250P strain with PtrA or hph.

Schematic of gene replacement design. To introduce the *srbA* L250P mutation, the *srbA* mutated gene and the A. PtrA cassette or hph cassette were PCR amplified, fused by overlap-PCR, and the final construct was used as a repair template. Transformation was performed with two guide crRNAs *srbA* 5’ and *srbA* 3'. B. Similar methodology was used to correct the mutated *srbA* L250P gene in the *Δcyp51A* B12 strain to a WT copy. C. ARMS-PCR analysis of one negative control isolate ("WT"), showing the control and "WT" bands, but not the "M" (Mutant) band, the *srbA* L250P repair template showing the control and "WT" bands, a *srbA* L250P mutant (the original evolved strain in which this mutation was originally found) showing the control and the "M" bands, and one of the isolates transformed with *srbA* L250P repair template (in this case, with hph, PtrA isolates not shown), showing the control and the "M" bands. Strains were subsequently verified by Sanger sequencing, D. a *srbA* sequence of "cCg" instead of "cTg" for the L250P, and E. a *srbA* sequence of "cTg" instead of "cCg" for the WT. E. Serial dilution assays of parental KU80 (WT), SrbA WT (control transformed with WT-*srbA*) , SrbA L250P independent isolates 1,2, and 3, in which the SrbA L250P mutation was inserted. Strains were grown on YAG agar containing increasing concentrations of VRC. The mutated strain selected for further study is highlighted in purple. F. Serial dilution assays of control evolved ΔCyp51A B12 (L250P), SrbA L250P (control transformed with SrbA L250P), SrbA fix independent isolates 1,2, and 3, in which WT SrbA was inserted into ΔCyp51A B12 (L250P) to replace SrbA L250P. Strains were grown on YAG agar containing increasing concentrations of VRC. The mutated strain selected for further study is highlighted in purple.

**Construction and verification of *ptaB* Q397frameshift and Q312stop strains.** Primers "PtaB-F" and "PtaB-R" (Table S3) were used for amplification of the mutated and WT *ptaB* gene, with 50 bp flanking 5' end of the gene, and primers "PtrA (PtaB)-F" and "PtrA (PtaB)-R" were used for amplification of the PtrA cassette, with 50 bp flanking 3' end of the *ptaB* gene. The two amplicons were then fused by overlap-PCR and the final constructs were used as a repair template (Figure S5.A). The amplicons were introduced into the *ΔKU80* strain, along with two gRNAs, one for each 5' and 3' ends of the target gene (Table S4), and Cas9 enzyme (IDT). Transformant colonies were screened on AMM + 0.1 μg/ml PtrA agar plates, after which selected colonies were streaked twice on YAG agar plates for strain purification. *ptaB* mutant isolates were selected by their white and fluffy phenotype (Figure S5.B). The final isolates of both Q397frameshift and Q312stop mutants were then verified by sequencing (Figure S5.C-D).


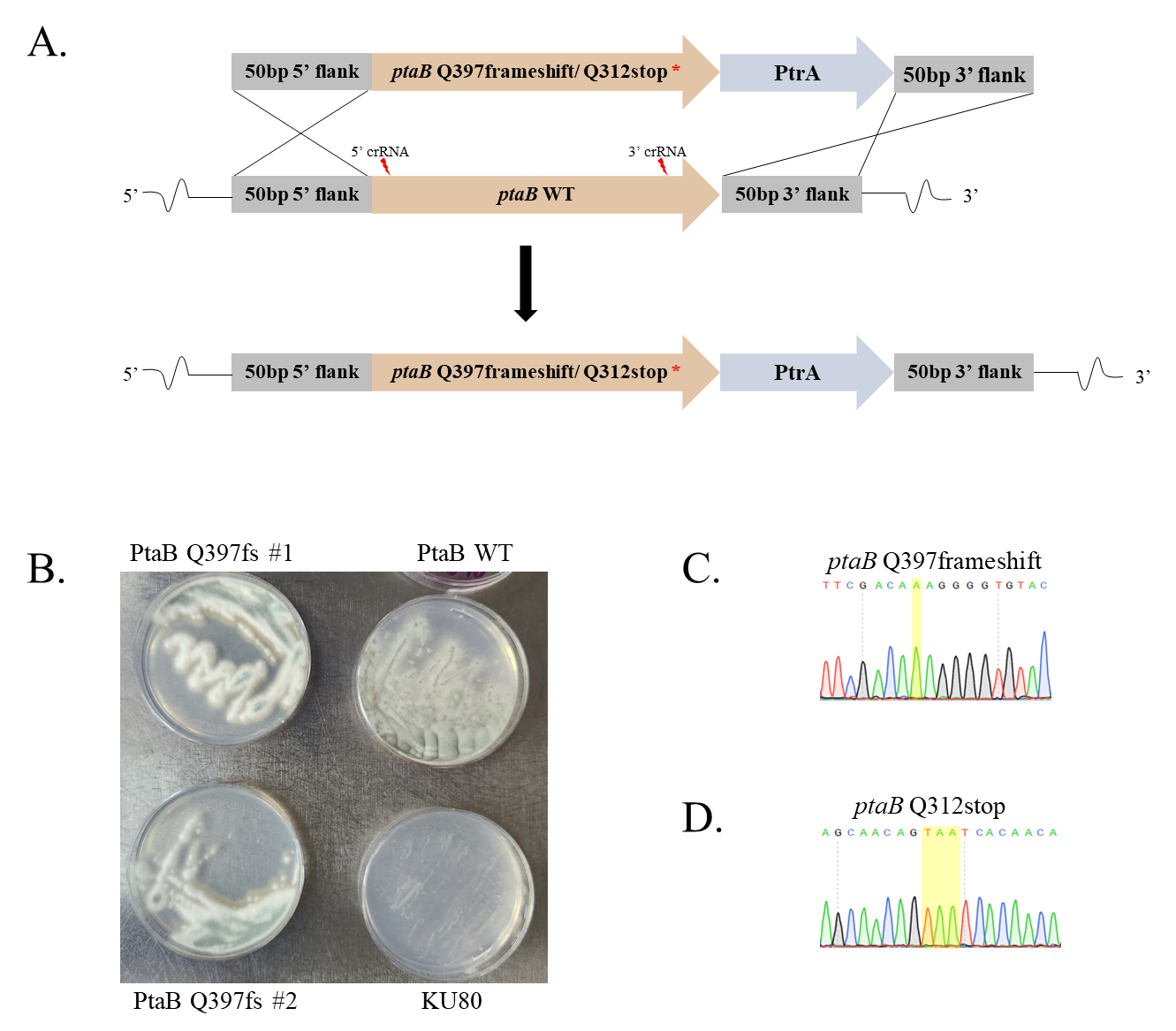


E.
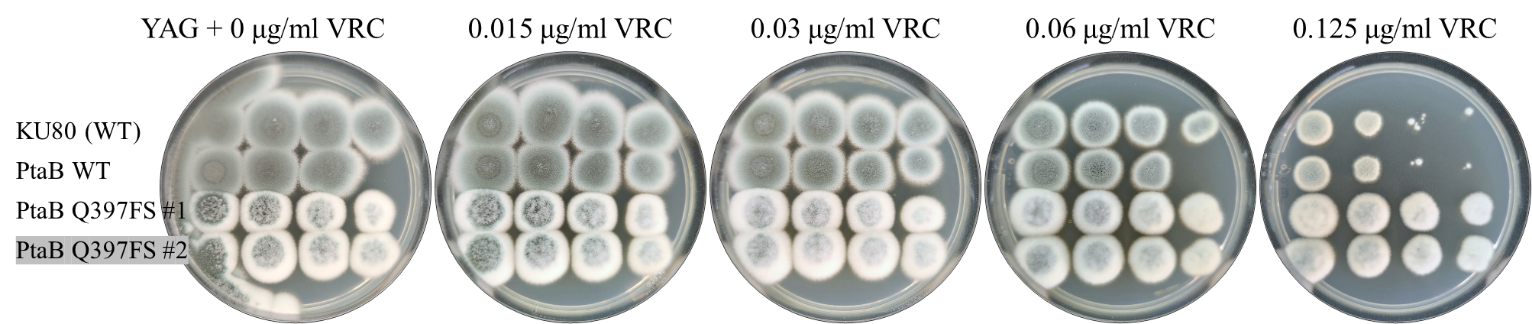


**Figure S5.** Generation and verification of *ptaB* Q397frameshift and Q312stop mutant strains.

Schematic of gene replacement design. A. To introduce the *ptaB* Q397frameshift and Q312stop mutations, the *ptaB* mutated gene and the PtrA cassette were PCR amplified, fused by overlap-PCR, and the final construct was used as a repair template. Transformation was performed with two guide crRNAs *ptaB* 5’ and *ptaB* 3'. B. The *ptaB* Q397frameshift (Q397fs) mutants have a fluffier and whiter phenotype compared to non-*ptaB* mutants, isolates that shared this phenotype were suspected as *ptaB* mutants of both Q397frameshift and Q312stop mutations. Strains were tested via isolation streaks on AMM + 0.1 μg/ml PtrA plates. Strains were subsequently verified by Sanger sequencing, C. An additional "A" in the *ptaB* sequence for the Q397frameshift, D. A *ptaB* sequence of "Taa" instead of "Caa" for the Q312stop. E. Serial dilution assays of parental KU80 (WT), PtaB WT (control transformed with WT-*ptaB*, PtaB Q397FS (frameshift) independent isolates 1 and 2, in which the PtaB Q397FS mutation was inserted. Strains were grown on YAG agar containing increasing concentrations of VRC. The mutated strain selected for further study is highlighted in grey.

**Supplementary Tables.**

| **Table S1.** Strains used in this study | | | |
| --- | --- | --- | --- |
| Strain | Mutations | Background | Source |
| *ΔKU80* (WT) |  | *ΔKU80* | Da Silva Ferreira et al., 2006 |
| WT Control 29 |  | *ΔKU80* | This study |
| WT A20 | *cyp51A* G448S | *ΔKU80* | This study |
| WT B29 | *cyp51B* G457S / *abcC* R339H / *erg25B* D253G | *ΔKU80* | This study |
| WT C12 | *erg25B* G178D / *ptaB* Q397frameshift | *ΔKU80* | This study |
| WT D12 | *erg25B* G178D / *ptaB* Q397frameshift | *ΔKU80* | This study |
| *Δcyp51A* | *Δcyp51A*-PtrA | *ΔKU80* | This study |
| *Δcyp51A*-hph | *Δcyp51A*-hph | *ΔKU80* | Handelman et al., 2021 |
| *Δcyp51A* Control 30 |  | *Δcyp51A*-hph | This study |
| *Δcyp51A* A3 |  | *Δcyp51A*-hph | This study |
| *Δcyp51A* B12 | *hmg1* G386W / *abcC* S570N / *ptaB* Q312stop / *srbA* L250P | *Δcyp51A*-hph | This study |
| *Δcyp51A* C30 | *cyp51B* G457S / *hmg1* F262del / *erg25B* K294frameshift | *Δcyp51A*-hph | This study |
| *Δcyp51A* D2 | *hmg1* L493P | *Δcyp51A*-hph | This study |
| Hmg1 G386W | *hmg1* G386W | *ΔKU80* | This study |
| Hmg1 L493P | *hmg1* L493P | *ΔKU80* | This study |
| AbcC R339H | AbcC R339H | *ΔKU80* | This study |
| Erg25B D253G-PtrA | *erg25B* D253G-PtrA | *ΔKU80* | This study |
| *Δerg25B* | *Δerg25B*-PtrA | *ΔKU80* | This study |
| PtaB Q397frameshift-PtrA | *ptaB* Q397frameshift-PtrA | *ΔKU80* | This study |
| *Δcyp51A* Hmg1 F262del | *hmg1* F262del | *Δcyp51A*-hph | This study |
| *Δcyp51A* Hmg1 L493P | *hmg1* L493P | *Δcyp51A*-hph | This study |
| *Δcyp51A* Hmg1 G386W | *hmg1* G386W | *Δcyp51A*-hph | This study |
| *Δcyp51A* B12 SrbA-fix | *hmg1* G386W / *abcC* S570N / *ptaB* Q312stop / *srbA* WT-PtrA | *Δcyp51A* B12 | This study |
| *ΔsrbA* | *ΔsrbA*-hph | *ΔKU80* | Prof. Michael Bromley, Manchester University |
| SrbA L250P-hph | *srbA* L250P-hph | *ΔKU80* | This study |
| SrbA L250P-PtrA | *srbA* L250P-PtrA | *ΔKU80* | This study |
| PtaB Q312stop | *ptaB* Q312stop-PtrA | *ΔKU80* | This study |
| SrbA-PtaB | *ptaB* Q312stop-PtrA / *srbA* L250P-hph | *ΔKU80* | This study |

| **Table S2**. Gene deletions and duplications identified in the VRC-evolved strains | |
| --- | --- |
| Strain | Deletions/Duplications |
| WT A20 | AFUB_017190 (partial duplication), AFUB_017200 (duplication), AFUB_048850 (partial  duplication) |
| WT B29 | AFUB_000030 (partial duplication), AFUB_017180 (duplication), AFUB_017190 (partial  duplication), AFUB_048820 (partial duplication), AFUB_048830 (partial duplication),  AFUB_048850 (duplication), intergenic region in DS499601 (partial duplication),  AFUB_086620 (duplication) |
| Δcyp51A A3 | AFUB_072250 (partial deletion) |
| Δcyp51A B12 | AFUB_048850 (partial deletion) |
| Δcyp51A C30 | AFUB_086620 (partial duplication) |

| **Table S3.** Amplification primers used in this study | | | |  |
| --- | --- | --- | --- | --- |
| Target gene | Primer name | Primer sequence (5' → 3') | Final construct | |
| *cyp51A* | Cyp51A-F | GGGCTGGAGATACTATGGCTTTCA | Mutated *cyp51A* G448S | |
|  | Cyp51A-R | CAGGTTTTCGCACGAGCTTC |  |  |
| *cyp51B* | Cyp51B-F | ATGGGTCTCATCGCGTTCATT | Mutated *cyp51*B G457S | |
|  | Cyp51B-R | TCAGGCTTTGGTAGCGGACTC |  |  |
| *hmg1* | Hmg1-F | CAGCATCGAGTCGAGAGAATTT | Mutated *hmg1* F262del or G386W or L493P | |
|  | Hmg1-R | CTGCGTTACTCGGTCTTGGTAC |  |  |
| *abcC* | AbcC-F | TCTCGTCCAAGCAGATGCGCAA | Mutated *abcC* R339H | |
|  | AbcC -R | ACGAAGCGGCCATCGTACGG |  |  |
| *ptaB* | PtaB-F | TCATGGCCCTCAGAACATCCAGCA | Mutated *ptaB* Q397frameshift or Q312stop with PtrA on the 3' end | |
|  | PtaB-R | GGATCCCGTAATCAATTGGCCTCTCATAGAAAGTGCGATCCAAG |  |  |
| ptrA | PtrA (PtaB)-F | CTTGGATCGCACTTTCTATGAGAGGCCAATTGATTACGGGATCC |  |  |
|  | PtrA (PtaB)-R | AAAGACCCCAAAAGATATCATTCCTCAGAATCATTGAGGAAAGCAAAAAGCCTAGATGGCCTCTTGCATC |  |  |
| *erg25B* | Erg25B KO-F | TTCAACACCCAGTTGTCTCATACTAGGATACAGGTGCGATATCAACTACAAGGCCAATTGATTACGGGATCC | PtrA with *erg25B* flanks | |
|  | Erg25B KO-R | ACCATAGAGCAATTGGCATGAGTCCTAAGCAGGACAGGTAAAGGCTGTCATCGATTTATGATAGCAGTGCCCTAGATGGCCTCTTGCATC |  |  |
| *erg25B* | Erg25B-F | ACGGTCCGGCGTGTTCAGTTAA | Mutated *erg25B* D253G with PtrA on the 3' end | |
|  | Erg25B-R | GGATCCCGTAATCAATTGGCCTTATATAGTGGCTTTATAGTCTG |  |  |
| ptrA | PtrA (Erg25B)-F | CAGACTATAAAGCCACTATATAAGGCCAATTGATTACGGGATCC |  |  |
|  | PtrA (Erg25B)-R | TAAGTTATATAATCTAATAGTCTGCACTGCAGCACTATCTGTGTTGGCTGCCTAGATGGCCTCTTGCATC |  |  |
| *srbA* | SrbA-F | TCTCCCGGGCGGAGATCTGAATACCAATGTATCCCC | Mutated *srbA* L250P or WT *srbA* with PtrA on the 3' end | |
|  | SrbA (PtrA)-R | ATCCCGTAATCAATTGGCCTGTATCATGCATTTGGAGGCA |  |  |
| ptrA | PtrA (SrbA)-F | TGCCTCCAAATGCATGATACAGGCCAATTGATTACGGGAT |  |  |
|  | PtrA (SrbA)-R | CTTCCCGATCCATGAAACCCTGCCACGTTACATCTGAGATCCATATACATCCTAGATGGCCTCTTGCATC |  |  |
| *srbA* | SrbA-F | TCTCCCGGGCGGAGATCTGAATACCAATGTATCCCC | Mutated *srbA* L250P with hph on the 3' end | |
|  | SrbA (hph)-R | ATTGGACTTCTGTACCTAGGGTATCATGCATTTGGAGGCA |  |  |
| hph | hph (SrbA)-F | TGCCTCCAAATGCATGATACCCTAGGTACAGAAGTCCAAT |  |  |
|  | hph (SrbA)-R | CTTCCCGATCCATGAAACCCTGCCACGTTACATCTGAGATCCATATACATTCTAGAAAGAAGGATTACCT |  |  |

| **Table S4**. crRNAs used in this study | | |
| --- | --- | --- |
| Target gene | 5' crRNA | 3' crRNA |
| *cyp51A* | GGTGCCGATGCTATGGCTTA | GAAGCCAAGCATCATCGGCT |
| *cyp51B* | ACATGGGTGCTTGTTGGAAT | AAAAGATCGGCCAAGCGGTT |
| *hmg1* | GCACCCTATACACACCATTG | GCATGGCGAAACATGAAGTA |
| *abcC* | GATGTGGTGATGGCCATGCT | AGGCATGTCACAGAGCATCG |
| *ptaB* | GGTTGAGGAGGGTTCGACTG | GACCGCCGTTGGCACACCAT |
| *erg25B* | ATTCTTCGGTCTGTCTACGT | AATTGGCATGAGTCCTAAGC |
| *srbA* | AGACAGTGTGCCTAGTCTAC | AGAGGTAACAATCTGATCAG |

| **Table S5**. ARMS-PCR primers used in this study | | | |
| --- | --- | --- | --- |
| Target gene | Mutation | Primer name | Primer sequence (5' → 3') |
| *cyp51A* | G448S | Cyp51A OF | TGCTGAGACTGGCCTCACAGC |
|  |  | Cyp51A WT-F | CACGTCAAGTCCCTATCTTCCGTGTG |
|  |  | Cyp51A M-R | ATACAGCGGTGTCGGCCAGCCCT |
|  |  | Cyp51A OR | GCCCTCGAGGGGCTGAATTAAGTAT |
| *cyp51B* | G457S | Cyp51B OF | CGTGTTCTCGGATCTGACTTG |
|  |  | Cyp51B WT-R | CAATGCACCTATGCCGTCCAGCTCC |
|  |  | Cyp51B M-F | CCAATAGCCCGTACCTCCCGTGTA |
|  |  | Cyp51B R | TCAGGCTTTGGTAGCGGACTC |
| *hmg1* | G386W | Hmg1-386-OF | TATGCTGCCATATTTGCTGATG |
|  |  | Hmg1-386-WT-R | GAGAAAACAGAATTGTCGCAATCC |
|  |  | Hmg1-386-M-F | CGCTTCTGGAGTCCAGGATT |
|  |  | Hmg1-386-OR | ACGAGACAGTAGAGGTAGGCC |
| *abcC* | R339H | AbcC-F | TCTCGTCCAAGCAGATGCGCAA |
|  |  | AbcC WT-R | TCGCGCTGTCCAGACAAC |
|  |  | AbcC M-F | GCTGGGACAACAGCACACA |
|  |  | AbcC OR | AAATCAGCGGTTGTCTGACGT |
| *ptaB* | Q312stop | PtaB OF | GCAACCGGGTCAGCATAC |
|  |  | PtaB WT-F | CAGCCCCAGCAACCGC |
|  |  | PtaB M-R | GGTTGCCCCTGTTGTGAGTA |
|  |  | PtaB OR | GAATCTCTCTCGCGCGCA |
| *erg25B* | D253G | Erg25B OF | CCATGATGTACCAGATAGCCATT |
|  |  | Erg25B WT-R | CGACAAATTTCTCGTGGTGAATAT |
|  |  | Erg25B M-F | CTGGAGCTGATCACCAGGG |
|  |  | Erg25B-R | ATGAACCTAGCAACCCCAGTCATCA |
| *srbA* | L250P | SrbA OF | TGTTCAAAGCTAAACAGGCGC |
|  |  | SrbA WT-F | TTGGAGGAAGAGAATACCGCTCT |
|  |  | SrbA M-R | ACTGACGTAATCGGATCTGCG |
|  |  | SrbA OR | TTCTGTGCCTAGGCCTTCAA |

| **Table S6**. qPCR primers used in this study | | |
| --- | --- | --- |
| Target gene | Forward primer | Reverse primer |
| *β-tubulin* | GGTAACTCCACCTCCATTCAG | AACTCCATCTCGTCCATACC |
| *cyp51A* | CCAGTACAAGGATCTTGACAAAC | ACCGGGAGGAATCATGTAAG |
| *cyp51B* | AGCAGAAGAAGTTCGTCAAATAC | TCGAAGACGCCCTTGTG |
| *erg1* | GATAATGCACCGATCCTCCTG | GCTCTTCACACCACCATTCT |
| *erg24A* | GTCTAGTGGCTCGAAGCTAATG | TGATGGTGTAGCCAGCAATC |
| *erg24B* | TCTATGTCCTTGATGCCCTTTAC | GACACTGAGGTATCTGGTTTGG |
| *erg25A* | GTCCTCTTCCACCCAATGA | GCCAGTAGTGGTATGTGTCT |
| *erg25B* | GGACCATGATGTACCAGATAGC | TACCAAAGGGTGCCGAATAC |
| *erg26B* | GCACCTCTGACAAGGACTAAA | GCAGTTCTTAACCCGTCCTTA |
| *erg3C* | CTCTGCCATACCAGTCAGTATC | GCAGCGAAGAGAACCATGTA |
| *erg7C* | CAGGTGACTAATACCGCCTATG | TTGCTGCTCCTTCACAAGATA |
| *hmg1* | CATCGAACATTGTCACTGCT | AAGGCATAGACACAGCGAT |
| *ptaB* | GTGGTCGCATTGTGGAAAG | CACGACAATGTCAAGCATCTC |
| *srbA* | CGGATTCCTTACCGAGTCATT | GTCATCGTCGGTTCCTTCTT |
